# Intratumoral and peritumoral radiomics based on contrast-enhanced MRI for preoperatively predicting treatment response of transarterial chemoembolization in hepatocellular carcinoma

**DOI:** 10.1186/s12885-023-11491-0

**Published:** 2023-10-24

**Authors:** Ying Zhao, Jian Zhang, Nan Wang, Qihao Xu, Yuhui Liu, Jinghong Liu, Qinhe Zhang, Xinyuan Zhang, Anliang Chen, Lihua Chen, Liuji Sheng, Qingwei Song, Feng Wang, Yan Guo, Ailian Liu

**Affiliations:** 1 Department of Radiology, The First Affiliated Hospital, Dalian Medical University, No. 222 Zhongshan Road, Xigang District, Dalian, Liaoning China; 2 Department of Interventional Radiology, The First Affiliated Hospital, Dalian Medical University, Dalian, China; 3 College of Medical Imaging, Dalian Medical University, Dalian, China; 4 GE Healthcare (China), Shanghai, China

**Keywords:** Hepatocellular carcinoma, Radiomics, Magnetic resonance imaging, Transarterial chemoembolization, Treatment response

## Abstract

**Background:**

Noninvasive and precise methods to estimate treatment response and identify hepatocellular carcinoma (HCC) patients who could benefit from transarterial chemoembolization (TACE) are urgently required. The present study aimed to investigate the ability of intratumoral and peritumoral radiomics based on contrast-enhanced magnetic resonance imaging (CE-MRI) to preoperatively predict tumor response to TACE in HCC patients.

**Methods:**

A total of 138 patients with HCC who received TACE were retrospectively included and randomly divided into training and validation cohorts at a ratio of 7:3. Total 1206 radiomics features were extracted from arterial, venous, and delayed phases images. The inter- and intraclass correlation coefficients, the spearman’s rank correlation test, and the gradient boosting decision tree algorithm were used for radiomics feature selection. Radiomics models on intratumoral region (TR) and peritumoral region (PTR) (3 mm, 5 mm, and 10 mm) were established using logistic regression. Three integrated radiomics models, including intratumoral and peritumoral region (T-PTR) (3 mm), T-PTR (5 mm), and T-PTR (10 mm) models, were constructed using TR and PTR radiomics scores. A clinical-radiological model and a combined model incorporating the optimal radiomics score and selected clinical-radiological predictors were constructed, and the combined model was presented as a nomogram. The discrimination, calibration, and clinical utilities were evaluated by receiver operating characteristic curve, calibration curve, and decision curve analysis, respectively.

**Results:**

The T-PTR radiomics models performed better than the TR and PTR models, and the T-PTR (3 mm) radiomics model demonstrated preferable performance with the AUCs of 0.884 (95%CI, 0.821–0.936) and 0.911 (95%CI, 0.825–0.975) in both training and validation cohorts. The T-PTR (3 mm) radiomics score, alkaline phosphatase, tumor size, and satellite nodule were fused to construct a combined nomogram. The combined nomogram [AUC: 0.910 (95%CI, 0.854–0.958) and 0.918 (95%CI, 0.831–0.986)] outperformed the clinical-radiological model [AUC: 0.789 (95%CI, 0.709–0.863) and 0.782 (95%CI, 0.660–0.902)] in the both cohorts and achieved good calibration capability and clinical utility.

**Conclusions:**

CE-MRI-based intratumoral and peritumoral radiomics approach can provide an effective tool for the precise and individualized estimation of treatment response for HCC patients treated with TACE.

**Supplementary Information:**

The online version contains supplementary material available at 10.1186/s12885-023-11491-0.

## Introduction

Hepatocellular carcinoma (HCC) is the most common liver malignancy and the third leading cause of death among various cancers [1]. Liver transplantation, resection, and ablation are the curative therapies for patients with early HCC [2]. Unfortunately, the majority of HCC patients are not suitable for curative treatment at the time of diagnosis because of poor liver function, multifocal disease, vascular involvement, and extrahepatic spread [3]. Transarterial chemoembolization (TACE) is widely used as a bridge to liver transplantation, or as the standard treatment for patients with intermediate HCC [4]. Nevertheless, the therapeutic efficacy of TACE varies greatly due to the high heterogeneity of HCC [5]. Several studies have evidenced that the overall response rates following TACE range from 15 to 85% and the cumulative rates of local tumor progression at 1, 3, and 5 years are 33%, 52%, and 73%, respectively [6, 7]. Therefore, it is crucial to preoperatively estimate tumor response to TACE treatment which may aid in guiding subsequent therapeutic strategies.

Magnetic resonance imaging (MRI)-based evaluations that are noninvasive and repeatable can be used to preoperatively assess treatment response. Several scholars have reported that larger lesion diameter, irregular margin, arterial peritumoral enhancement, satellite nodule, and apparent diffusion coefficient (ADC) value are associated with therapeutic efficacy of TACE treatment [8–11]. Although these imaging characteristics are encouraging, they are not sufficient for the individual evaluation of tumor response to TACE, and the ability to predict TACE efficacy in HCC is limited when a high degree of tumor heterogeneity.

Radiomics, an emerging and non-invasive approach, can extract high-throughput quantitative data from multi-modality imaging and characterize tumor heterogeneity, which may potentially guide individual medicine [12, 13]. Numerous studies have demonstrated that radiomics-based models effectively identify the diagnosis and pathological characteristics or predict therapeutic efficacy and prognosis of cancer patients for clinical decision-making [14–17]. Recently, there has been increasing interest in evaluating radiomics patterns of the region surrounding the visible tumor [15, 17–19]. Recurrence or metastasis of HCC is mainly intrahepatic, indicating that the peritumoral liver tissue may be a favorable soil for the spreading hepatoma cells [20]. Several scholars have reported that HCC patients with microvascular invasion (MVI), epithelial cell adhesion molecule (EpCAM), programmed death ligand 1 (PD-L1) expression, and higher CD68 + cell density in peritumoral tissues have a significantly higher risk of recurrence or metastasis and cancer-related death [21–24]; thus, peritumoral tissues might have valuable predictive information of HCC prognosis. Several recent studies have reported that CT or MRI-based radiomics on intratumoral and peritumoral regions can effectively predict MVI, vessels encapsulating tumor clusters (VETC), anti-PD-1 treatment efficacy, and prognosis of resection or TACE in patients with HCC, which may achieve an enhanced prediction of the individualized risk estimation [15, 17–19, 25–27]. However, the value of intratumoral and peritumoral radiomics based on MRI in predicting treatment response of HCC after TACE remains unknown.

Therefore, the present study aimed to determine whether radiomics assessment of HCC peritumoral regions based on contrast-enhanced MR (CE-MR) images could provide valuable information about TACE response and enhance the ability of intratumoral radiomics for the prediction of treatment efficacy of TACE in patients with HCC.

## Materials and methods

### Patients

This retrospective study was approved by the Institutional Review Board of the First Affiliated Hospital of Dalian Medical University and the requirement for informed consent was waived due to the retrospective nature of the study.

**Fig. 1:**
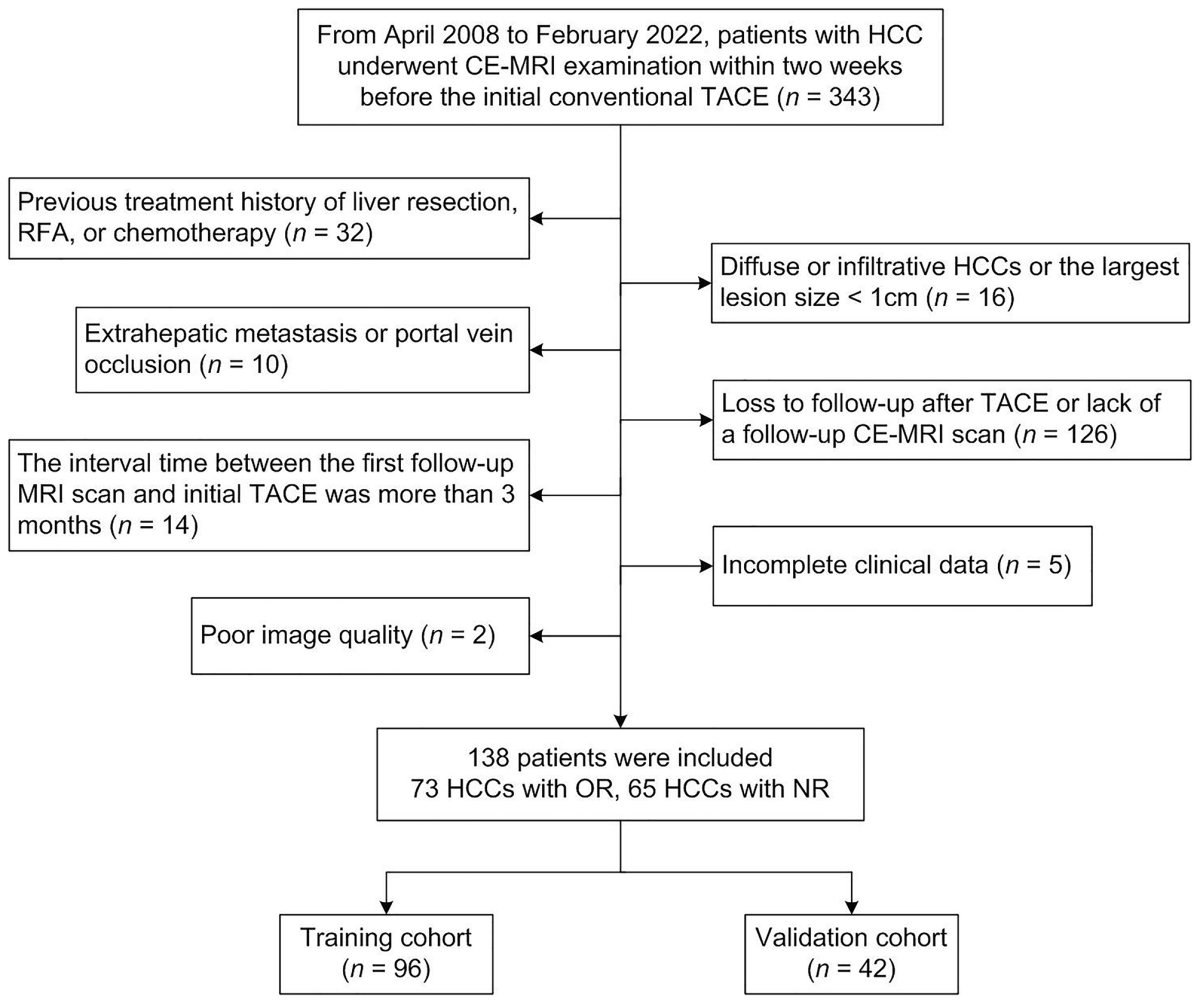
Flowchart of patient recruitment

From April 2008 to February 2022, 343 consecutive patients with HCC who underwent CE-MRI examination before conventional TACE at our institution were recruited. The diagnosis of HCC was confirmed by histopathology or non-invasive criteria defined by the American Association for the Study of Liver Disease (AASLD) based on specific imaging features [28]. The inclusion criteria were: (1) received TACE as the first-line therapy; (2) underwent CE-MRI examination within two weeks before therapy. The exclusion criteria were: (1) previous oncological treatment, including liver resection, radiofrequency ablation (RFA), or chemotherapy (*n* = 32); (2) diffuse or infiltrative HCCs or the largest lesion size < 1 cm (*n* = 16); (3) extrahepatic metastasis or portal vein occlusion (*n* = 10); (4) loss to follow-up after TACE or lack of a follow-up CE-MRI scan (*n* = 126); (5) the interval time between the first follow-up MRI scan and initial TACE was more than 3 months (*n* = 14); (6) incomplete clinical data (*n* = 5); (7) poor image quality (*n* = 2). Fig. 1 shows the flowchart of patient recruitment, and 138 patients were enrolled in this study and randomly divided into a training cohort (*n* = 96) and a validation cohort (*n* = 42) at a ratio of 7:3. Of the 138 HCC patients described above, 64 patients were included between April 2008 and June 2015, and the other 74 patients were enrolled between July 2015 and February 2022.

Pretherapeutic clinical characteristics including age, gender, history of hepatitis B or C, alpha-fetoprotein (AFP), alanine aminotransferase (ALT), aspartate aminotransferase (AST), γ-glutamyltranspeptadase (GGT), alkaline phosphatase (ALP), total bilirubin (TBIL), albumin (ALB), platelet count (PLT), prothrombin time (PT), Child-Pugh class, Eastern Cooperative Oncology Group (ECOG) performance status, and Barcelona Clinic Liver Cancer (BCLC) stage for each patient were retrospectively collected within 1 week before TACE.

### MRI protocol

All MRI examinations were performed with 1.5T or 3.0T MR scanner (Signa, HDXT, GE Healthcare) with a phased-array 8-channel sensitivity encoding abdominal coil. CE-MRI examination was performed using the liver acquisition with volume acceleration (LAVA) protocol with fat-suppressed T1-weighted 3D fast-spoiled gradient-recalled echo sequence. The contrast enhanced images consisted of arterial phase (AP), portal venous phase (PVP), and delayed phase (DP) images, which were obtained at 40 s, 70 s, and 90 s, respectively, after the start of contrast injection of Gd-diethylenetriamine pentaacetic acid (Gd-DTPA) (Bayer Schering Pharma AG, Germany) at a patient weight-dependent dose of 0.1 mmol/kg with an injection rate of 2.5 mL/s through a median cubital vein. Other MRI sequences were also performed, including in- and opposed-phase fast-spoiled gradient-recalled echo T1-weighted imaging (T1WI) and fat-suppressed fast spin-echo T2-weighted imaging (T2WI). The detailed scanning parameters are shown in Supplementary Data S1.

### Image analysis

Two radiologists (reader 1, Y.Z. and reader 2, N.W., with 8-year and 7-year experience in abdominal MRI) independently analyzed pretherapeutic MR images, and they were aware of the diagnosis of HCC but blinded to clinical information and imaging report. The radiologists evaluated the following imaging traits for each patient: (1) tumor size; (2) tumor number; (3) tumor margin; (4) intratumoral necrosis; (5) intratumoral hemorrhage; (6) intratumoral fat; (7) tumor encapsulation; (8) arterial peritumoral enhancement; (9) satellite nodule; (10) internal arteries; (11) radiological cirrhosis. Tumor diameter was recorded as mean value, and any discrepancy in imaging feature assessment was resolved by means of reevaluation by another senior radiologist (J.H.L., with 20-year experience in abdominal MRI). Detailed definitions of these imaging features and representative MR images are listed in Supplementary Data S2.

### TACE Procedure and Treatment Response Assessment

All conventional TACE procedures were carried out by interventional radiologists with no less than 10 years of clinical experience. The detailed description of TACE procedure is shown in Supplementary Data S3. All HCC patients were regularly monitored for therapeutic effect via contrast CT or CE-MRI within 1–3 months after the initial TACE, and then every 3–4 months thereafter. The modified Response Evaluation Criteria in Solid Tumors (mRECIST 1.1) criterion was utilized to assess the tumor response in patients with HCC based on pre- and post-therapeutic arterial MR images. Tumor response was classified into four categories according to the mRECIST system as follows: complete response (CR), partial response (PR), stable disease (SD), and progression disease (PD) [29]. In the present study, all patients were divided into the objective response (OR) group (CR and PR patients) and the non-response (NR) group (SD and PD patients). Visualization of tumor response assessment is showed in Supplementary Data S4.

### Tumor segmentation and Radiomics feature extraction

Pretherapeutic CE-MR images were exported from the picture archiving and communication system (PACS) and then used for tumor segmentation and radiomics feature extraction. The AK software (Artificial Intelligence Kit, Version 3.2.5, GE Healthcare) was used to process the images before segmenting the tumor. AP, PVP, and DP images were resampled to a uniform voxel size of 1 × 1 × 1 mm via linear interpolation algorithm to standardize the voxel spacing [17]. Intensity normalization of images was performed to correct the scanner effect. Tumor segmentation was performed by manually delineating the region of interest (ROI) along the tumor contour on each axial slice of AP, PVP, and DP images using an open-source software (ITK-SNAP, version 3.6.0, http://www.itksnap.org/). The ROI was required to include capsule surrounding the tumor and to exclude tumor surrounding vessels, and then every ROI was automatically merged into volume of interest (VOI). Notably, in terms of multifocal HCCs, the largest nodule with abundant vascularity was selected as the delineated lesion and used for subsequent radiomics analysis [18, 30]. To capture radiomics features from the tumor periphery, the VOIs of peritumoral region (PTR) were generated by automatically expanding 3 mm, 5 mm, and 10 mm from the lesion border using AK software. If the ROI was beyond the parenchyma of the liver after the expansion, the portion beyond the parenchyma was removed manually.

**Fig. 2:**
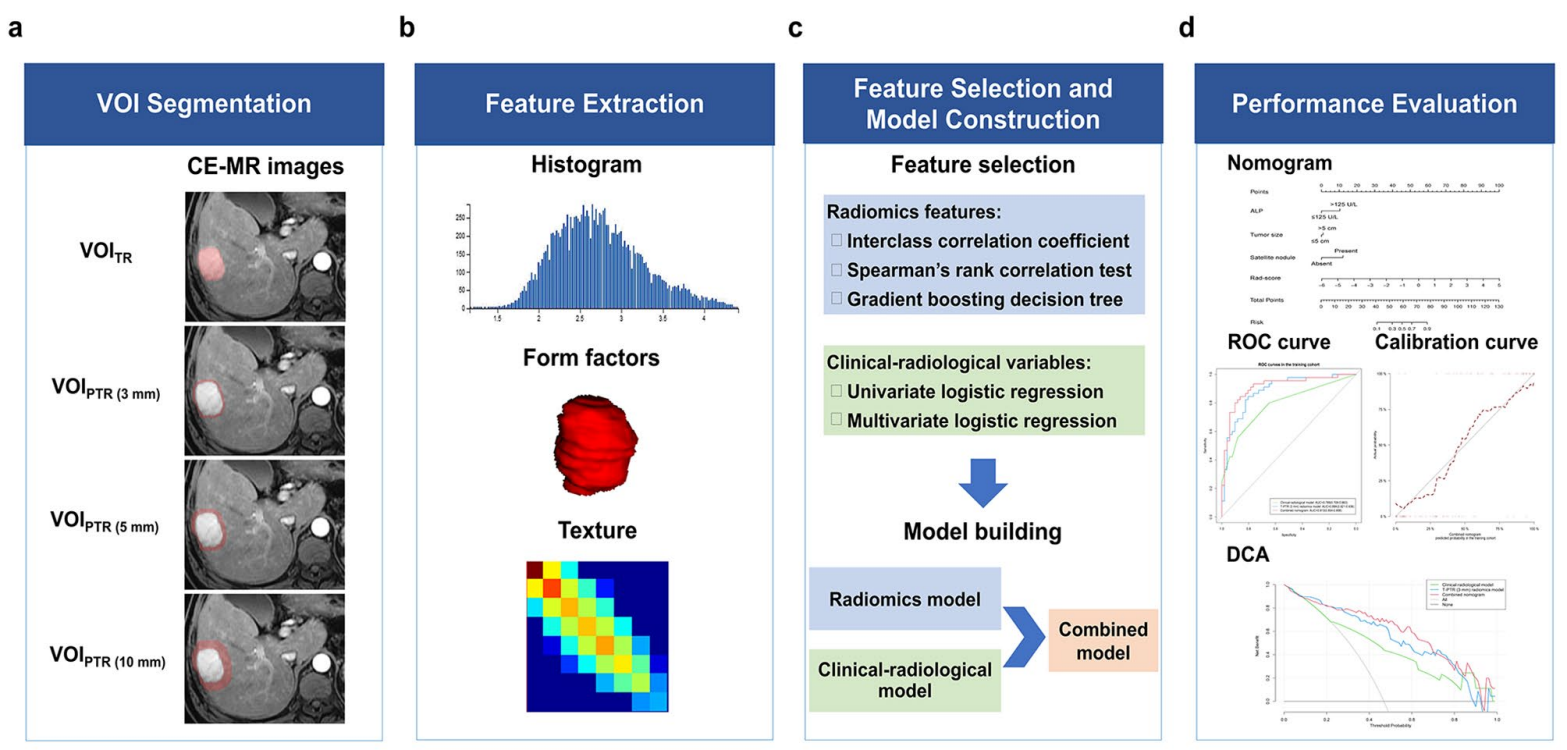
The workflow of radiomics analysis in the present study. (**a**) Contrast-enhanced MR imaging was acquired. Tumors were manually delineated around the entire tumor outline on each axial slice of arterial phase (AP), portal venous phase (PVP), and delayed phase (DP) images, and peritumoral expansion (3 mm, 5 mm, and 10 mm) were automatically generated. (**b**) Total 1206 radiomics features on enhanced images were extracted. (**c**) Three steps of feature dimensionality reduction were applied for all extracted features. Seven radiomics models based on intratumoral region (TR), peritumoral region (PTR), and TR combined PTR radiomics scores were constructed using logistic regression analysis. A clinical-radiological model and a combined model incorporating the optimal radiomics score and clinical-radiological independent risk factors were constructed. (**d**) The combined nomogram was presented to provide a more understandable treatment response measurement for individualized evaluation, followed by receiver operating curve analysis, calibration curve, and decision curve analysis

Radiomics features were extracted using AK software for intratumoral region (TR), PTR (3 mm), PTR (5 mm), and PTR (10 mm). A total of 1206 radiomics features were extracted for each VOI based on AP, PVP, and DP images. The extracted radiomics features included: 42 histograms, 15 form factors, 10 Haralick features, 144 grey level co-occurrence matrix (GLCM) with an offset of 1/4/7, 180 grey level run length matrix (GLRLM) with an offset of 1/4/7, and 11 grey-level zone size matrix (GLZSM). Details of radiomics features are listed in Supplementary Data S5. Z-score normalization of radiomics features was performed to reduce the bias caused by different dimensions. The workflow of the radiomics analysis is depicted in Fig. 2.

Inter- and intraclass correlation coefficients (ICCs) were used to assess the reproducibility of each radiomics feature extracted from 30 randomly chosen patients. To assess the interobserver reproducibility, the ROI delineation was performed by two abdominal radiologists (readers 1 and 2, Y.Z. and N.W.) independently who were blinded to all patients’ information. To evaluate the intraobserver reproducibility, reader 1 repeated the ROI delineation and feature extraction at a 1-month interval. Finally, reader 1 completed the remaining image segmentation and feature extraction.

### Feature selection and Radiomics Model Construction

To remove potentially redundant features and decrease data dimensions, we followed a three-step procedure to identify the most predictive radiomics features. First, the radiomics features with ICC values of both interobserver and intraobserver greater than 0.80 indicated satisfactory reproducibility and high robustness and were selected for further analysis. Second, the redundant features with correlation coefficients higher than 0.90 were eliminated following by the Spearman’s rank correlation test. Finally, the gradient boosting decision tree (GBDT) algorithm was applied to determine the top-ranked and most valuable features for predicting the tumor response. GBDT was proposed as a tree-based algorithm based on a greedy strategy (called gradient boosting) that evaluated the importance of a texture feature through the time it used as branching point for the tree [14]. After those steps, TR, PTR (3 mm), PTR (5 mm), and PTR (10 mm) radiomics models were separately established using logistic regression algorithm with 5-fold cross-validation. The radiomics score (Rad-score) was calculated for each patient using a linear combination of the selected features that were weighted by their respective coefficients. Finally, three integrated radiomics models, including intratumoral and peritumoral region (T-PTR) (3 mm), T-PTR (5 mm), and T-PTR (10 mm) radiomics models, were generated by logistic regression using TR rad-score and corresponding PTR rad-score. The optimal radiomics model with the highest area under the curve (AUC) was selected for further analysis.

### Clinical-radiological model construction

Univariate analysis was used to identify the significant variables among clinical-radiological characteristics associated with treatment response of HCC (*P* < 0.05). Multivariate logistic regression analysis was performed to identify the independent risk factors for predicting tumor response. Odds ratio and 95% confidence interval (CI) were calculated for each risk factor. The clinical-radiological model was constructed using the chosen independent risk factors.

## Combined Model Building and Nomogram Construction

A combined model integrating clinical-radiological risk factors and the optimal rad-score was constructed using the proposed logistic regression method. To provide an individual predictive graphical presentation, the combined model was presented as a nomogram. The nomogram could help calculate the predicted probability of NR for each individual patient. In addition, the most recent patients between March 2022 and April 2023 were used as an independent testing cohort to validate the performance of predictive models constructed by the training cohort (described in Supplementary Data S6).

### Statistical analysis

The Student’s *t*-test or Mann-Whitney *U*-test were used to compare the continuous variables between the OR and NR groups as appropriate. The chi-squared test or Fisher’s exact test were used to assess the categorical variables as appropriate. Interobserver agreement evaluation of the radiological features was qualified by Cohen kappa coefficient. Kappa values of 0.81–1.00 indicated excellent agreement, 0.61–0.80 signified substantial agreement, and 0.41–0.60 denoted moderate agreement. Receiver operating characteristic curve (ROC) analysis was performed to evaluate the performance of each predictive model. The AUC, accuracy, sensitivity, and specificity were calculated. We compared the predictive performance between different models using the Delong’s test. We also performed stratified analysis on the subgroups of inclusion time of predictive models. We used ROC analysis and AUC to evaluate the performance of each model on the subpopulations. Calibration curves and Hosmer-Lemeshow test were used to evaluate the degree of deviation between the predictions and actual outcomes. Decision curve analysis (DCA) was performed to validate the clinical utility of the nomogram. All statistical analyses for the present study were performed with R software (version 3.6.1, http://www.R-project.org). *P* < 0.05 was considered statistically significant.

## Results

### Patient characteristics

In total, 138 patients (mean age, 60.24 ± 8.91 years; range, 40–83 years; 115 male) were enrolled in this study. Among the 138 patients with HCC, 6 patients were determined by histopathology and 132 patients were identified by specific imaging features according to the AASLD guidelines. On the basis of the mRECIST criterion, the patients for CR, PR, SD, and PD were 13 (9.4%), 60 (43.5%), 51 (37.0%), and 14 (10.1%), respectively. All patients underwent post-therapeutic MRI examination following by the initial TACE treatment, with a median interval time of 44 days (range, 28–90 days) between the first TACE and the follow-up MRI examination. The clinical-radiological data in the OR and NR groups are summarized in Table 1. No significant difference was found in the clinical-radiological characteristics, except for tumor encapsulation between the training and validation cohorts.

**Table 1:** Patient clinical-radiological characteristics

Characteristics	Training cohort (*n* = 96)			Validation cohort (*n* = 42)			*P* value
	OR group (*n* = 51)	NR group (*n* = 45)	*P* value	OR group (*n* = 22)	NR group (*n* = 20)	*P* value	
Age (years, mean ± SD)	60.29 ± 8.41	60.42 ± 10.25	0.947	60.18 ± 9.02	59.75 ± 7.31	0.866	0.820
Gender (*n*, [%])			0.410			0.890	1.000
Male	44(86.3)	36(80.0)		18(81.8)	17(85.0)		
Female	7(13.7)	9(20.0)		4(18.2)	3(15.0)		
History of hepatitis B or C (*n*, [%])			0.541			0.361	0.410
Positive	38(74.5)	31(68.9)		19(86.4)	14(70.0)		
Negative	13(25.5)	14(31.1)		3(13.6)	6(30.0)		
AFP (IU/ml) (*n*, [%])			0.050			0.231	0.523
≤ 400	40(78.4)	27(60.0)		16(72.7)	11(55.0)		
> 400	11(21.6)	18(40.0)		6(27.3)	9(45.0)		
ALT (U/L) (*n*, [%])			0.521			0.827	0.904
≤ 50	36(70.6)	29(64.4)		15(68.2)	13(65.0)		
> 50	15(29.4)	16(35.6)		7(31.8)	7(35.0)		
AST (U/L) (*n*, [%])			0.024			0.187	0.302
≤ 40	31(60.8)	17(37.8)		11(50.0)	6(30.0)		
> 40	20(39.2)	28(62.2)		11(50.0)	14(70.0)		
GGT (U/L) (*n*, [%])			0.004			0.016	0.639
≤ 60	26(51.0)	10(22.2)		11(50.0)	3(15.0)		
> 60	25(49.0)	35(77.8)		11(50.0)	17(85.0)		
ALP (U/L) (*n*, [%])			< 0.001			0.005	0.313
≤ 125	46(90.2)	26(57.8)		19(86.4)	9(45.0)		
> 125	5(9.8)	19(42.2)		3(13.6)	11(55.0)		
TBIL (umol/L) (*n*, [%])			0.650			0.746	0.177
≤ 19	34(66.7)	28(62.2)		11(50.0)	11(55.0)		
> 19	17(33.3)	17(37.8)		11(50.0)	9(45.0)		
ALB (g/L) (*n*, [%])			0.897			0.789	0.922
< 40	29(56.9)	25(55.6)		13(59.1)	11(55.0)		
≥ 40	22(43.1)	20(44.4)		9(40.9)	9(45.0)		
PLT (×10^9^/L) (*n*, [%])			0.031			0.516	0.783
< 125	27(52.9)	14(31.1)		11(50.0)	8(40.0)		
≥ 125	24(47.1)	31(68.9)		11(50.0)	12(60.0)		
PT (s) (*n*, [%])			0.339			0.569	0.192
≤ 13	34(66.7)	34(75.6)		14(63.6)	11(55.0)		
> 13	17(33.3)	11(24.4)		8(36.4)	9(45.0)		
Child-Pugh class (*n*, [%])			0.585			0.379	0.078
A	44(86.3)	37(82.2)		17(77.3)	13(65.0)		
B	7(13.7)	8(17.8)		5(22.7)	7(35.0)		
ECOG performance status (*n*, [%])			0.060			0.095	0.084
0	49(96.1)	37(82.2)		20(90.9)	13(65.0)		
1	2(3.9)	8(17.8)		2(9.1)	7(35.0)		
BCLC stage (*n*, [%])			0.003			0.076	0.480
A	35(68.6)	18(40.0)		13(59.1)	8(40.0)		
B	13(25.5)	12(26.7)		7(31.8)	3(15.0)		
C	3(5.9)	15(33.3)		2(9.1)	9(45.0)		
Tumor size (*n*, [%])			< 0.001			0.001	0.936
≤ 5 cm	36(70.6)	15(33.3)		17(77.3)	5(25.0)		
> 5 cm	15(29.4)	30(66.7)		5(22.7)	15(75.0)		
Tumor number (*n*, [%])			0.664			0.275	1.000
Unifocal	35(68.6)	29(64.4)		13(59.1)	15(75.0)		
Multifocal	16(31.4)	16(35.6)		9(40.9)	5(25.0)		
Tumor margin (*n*, [%])			0.541			0.031	0.245
Smooth	38(74.5)	31(68.9)		17(77.3)	9(45.0)		
Non-smooth	13(25.5)	14(31.1)		5(22.7)	11(55.0)		
Intratumoral necrosis (*n*, [%])			0.005			0.129	0.833
Present	16(31.4)	27(60.0)		7(31.8)	11(55.0)		
Absent	35(68.6)	18(40.0)		15(68.2)	9(45.0)		
Intratumoral hemorrhage (*n*, [%])			0.030			0.060	0.611
Present	13(25.5)	21(46.7)		4(18.2)	9(45.0)		
Absent	38(74.5)	24(53.3)		18(81.8)	11(55.0)		
Intratumoral fat (*n*, [%])			0.112			0.872	0.826
Present	7(13.7)	12(26.7)		5(22.7)	4(20.0)		
Absent	44(86.3)	33(73.3)		17(77.3)	16(80.0)		
Tumor encapsulation (*n*, [%])			0.286			0.032	0.026
Present	39(76.5)	30(66.7)		15(68.2)	7(35.0)		
Absent	12(23.5)	15(33.3)		7(31.8)	13(65.0)		
Arterial peritumoral enhancement (*n*, [%])			0.050			0.118	0.846
Present	11(21.6)	18(40.0)		4(18.2)	8(40.0)		
Absent	40(78.4)	27(60.0)		18(81.8)	12(60.0)		
Satellite nodule (*n*, [%])			0.001			0.670	0.831
Present	1(2.0)	11(24.4)		2(9.1)	2(10.0)		
Absent	50(98.0)	34(75.6)		20(90.9)	18(90.0)		
Internal arteries (*n*, [%])			0.046			0.031	0.464
Present	18(35.3)	25(55.6)		5(22.7)	11(55.0)		
Absent	33(64.7)	20(44.4)		17(77.3)	9(45.0)		
Radiological cirrhosis (*n*, [%])			0.030			0.372	0.719
Present	36(70.6)	22(48.9)		14(63.6)	10(50.0)		
Absent	15(29.4)	23(51.1)		8(36.4)	10(50.0)		

OR, objective response; NR, non-response; SD, standard deviation; AFP, alpha-fetoprotein; ALT, alanine aminotransferase; AST, aspartate aminotransferase; GGT, γ-glutamyltranspeptadas; ALP, alkaline phosphatase; TBIL, total bilirubin; ALB, albumin; PLT, platelet count; PT, prothrombin time; ECOG, Eastern Cooperative Oncology Group; BCLC, Barcelona Clinic Liver Cancer

### Radiomics Model Development and evaluation

After inter- and intraobserver reproducibility analysis, the dimensions of feature spaces were 853 for VOI_TR_, 424 for VOI_PTR (3 mm)_, 452 for VOI_PTR (5 mm)_, and 716 for VOI_PTR (10 mm)_, respectively. Spearman’s rank correlation test allowed the selection of 100, 35, 43, and 90 features, respectively. GBDT revealed that the radiomics feature numbers ultimately consisted of 25, 14, 17, and 21 from the VOI_TR_, VOI_PTR (3 mm)_, VOI_PTR (5 mm)_, and VOI_PTR (10 mm)_, respectively, and were used for radiomics model building (TR, PTR (3 mm), PTR (5 mm), and PTR (10 mm) models). Finally, three T-PTR radiomics models based on TR and PTR rad-scores were constructed. The calculation formulae for the rad-score are shown in Supplementary Data S7.

The radiomics models demonstrated favorable discrimination in the both cohorts (AUC: training cohort, 0.810–0.892; validation cohort, 0.793–0.911). In the training cohort, the PTR (10 mm) radiomics model showed comparable performance compared with the TR model [AUC: 0.852 (95%CI, 0.785–0.911) vs. 0.836 (95%CI, 0.763–0.903)]. In the validation cohort, the TR, PTR (3 mm), PTR (5 mm) radiomics models showed equivalent performance with the AUCs of 0.820 (95% CI, 0.705–0.917), 0.823 (95% CI, 0.701–0.927), and 0.823 (95% CI, 0.711–0.924), respectively. Compared with TR and PTR radiomics models, the T-PTR radiomics models (T-PTR (3 mm), T-PTR (5 mm), and T-PTR (10 mm) models) performed better in predicting tumor response (∆AUC: training cohort, 0.031–0.082; validation cohort, 0.072–0.118). The (T-PTR) (3 mm) radiomics model demonstrated preferable performance with the AUCs of 0.884 (95%CI, 0.821–0.936) and 0.911 (95%CI, 0.825–0.975) in both training and validation cohorts. Morever, the (T-PTR) (3 mm) radiomics model also possessed high accuracy, sensitivity, and specificity of 0.812, 0.822, and 0.804, respectively, in the training cohort, and of 0.810, 0.800, and 0.818, respectively, in the validation cohort. Thus, we selected the T-PTR (3 mm) model as the best-performing radiomics model for further analysis. ROC curves and discrimination performance of the seven radiomics models in the two cohorts are shown in Supplementary Data S8 and Table 2.

**Table 2:** Discrimination performance of predictive models in the training and validation cohorts

Model		AUC (95% CI)	Accuracy	Sensitivity	Specificity	*P* value
Clinical-radiological model	TC	0.789 (0.709–0.863)	0.729	0.556	0.882	0.935
	VC	0.782 (0.660–0.902)	0.714	0.600	0.818	
TR model	TC	0.836 (0.763–0.903)	0.771	0.778	0.765	0.840
	VC	0.820 (0.705–0.917)	0.714	0.750	0.682	
PTR (3 mm) model	TC	0.817 (0.740–0.881)	0.750	0.889	0.627	0.940
	VC	0.823 (0.701–0.927)	0.714	0.800	0.636	
PTR (5 mm) model	TC	0.810 (0.739–0.880)	0.771	0.800	0.745	0.877
	VC	0.823 (0.711–0.924)	0.714	0.900	0.545	
PTR (10 mm) model	TC	0.852 (0.785–0.911)	0.760	0.822	0.706	0.468
	VC	0.793 (0.673–0.903)	0.762	0.900	0.636	
T-PTR (3 mm) model	TC	0.884 (0.821–0.936)	0.812	0.822	0.804	0.633
	VC	0.911 (0.825–0.975)	0.810	0.800	0.818	
T-PTR (5 mm) model	TC	0.883 (0.822–0.934)	0.823	0.733	0.902	0.661
	VC	0.909 (0.817–0.982)	0.810	0.650	0.955	
T-PTR (10 mm) model	TC	0.892 (0.834–0.942)	0.844	0.800	0.882	0.945
	VC	0.895 (0.815–0.964)	0.786	0.750	0.818	
Combined nomogram	TC	0.910 (0.854–0.958)	0.844	0.822	0.863	0.891
	VC	0.918 (0.831–0.986)	0.857	0.800	0.909	

TR, intratumoral region; PTR, peritumoral region; T-PTR, intratumoral and peritumoral region; TC, training cohort; VC, validation cohort; AUC, area under the curve; CI, confidence interval

### Clinical-radiological Model Development and evaluation

Interobserver agreements on the radiological features were substantial to excellent (kappa-value range: 0.772–1.000). Univariate and multivariate analyses of clinical-radiological characteristics for predicting treatment response in the training cohort are shown in Table 3. The univariate analysis indicated that AST, GGT, ALP, PLT, ECOG, BCLC stage, tumor size, intratumoral necrosis, intratumoral hemorrhage, satellite nodule, internal arteries, and radiological cirrhosis were significant clinical-radiological factors for discriminating the OR and NR groups in the training cohort (all *P* < 0.05). The multivariate logistic regression analysis demonstrated that ALP (odd ratio = 5.744; 95% CI: 1.780–18.532; *P* = 0.003), tumor size (odd ratio = 3.005; 95% CI: 1.154–7.826; *P* = 0.024), and satellite nodule (odd ratio = 9.865; 95% CI: 1.101–88.370; *P* = 0.041) were the independent risk factors for predicting NR in HCC patients. The clinical-radiological model was built by incorporating these three variables. The clinical-radiological model yielded the AUCs of 0.789 (95% CI, 0.709–0.863) and 0.782 (95% CI, 0.660–0.902) in the two cohorts, respectively (shown in Table 2).

**Table 3:** Univariate and multivariate analyses of clinical-radiological characteristics for predicting treatment response

Variables	Univariate analysis		Multivariate analysis	
	Odd ratio (95% CI)	*P* value	Odd ratio (95% CI)	*P* value
AST	2.553 (1.120–5.820)	0.026	—	—
GGT	3.640 (1.492–8.880)	0.005	—	—
ALP	6.723 (2.246–20.122)	< 0.001	5.744 (1.780–18.532)	0.003
PLT	2.491 (1.079–5.753)	0.033	—	—
ECOG	5.297 (1.062–26.428)	0.042	—	—
BCLC stage	2.716 (1.515–4.872)	< 0.001	—	—
Tumor size	4.800 (2.023–11.392)	< 0.001	3.005 (1.154–7.826)	0.024
Intratumoral necrosis	3.281 (1.417–7.600)	0.006	—	—
Intratumoral hemorrhage	2.558 (1.082–6.044)	0.032	—	—
Satellite nodule	16.176 (1.996–131.069)	0.009	9.865 (1.101–88.370)	0.041
Internal arteries	2.292 (1.007–5.213)	0.048	—	—
Radiological cirrhosis	0.399 (0.172–0.923)	0.032	—	—

The clinical-radiological characteristics with *P* value less than 0.05 in the univariate analysis are listed in the table. AST, aspartate aminotransferase; GGT, γ-glutamyltranspeptadase; ALP, alkaline phosphatase; PLT, platelet count; ECOG, Eastern Cooperative Oncology Group; BCLC, Barcelona Clinic Liver Cancer; CI, confidence interval

### Combined model and Nomogram Development and evaluation

**Fig. 3:**
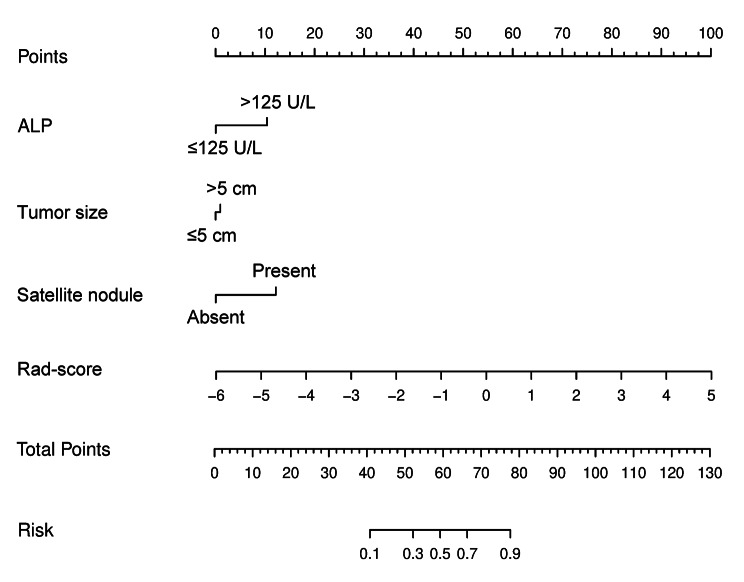
The combined nomogram incorporated alkaline phosphatase (ALP), tumor size, satellite nodule, and radiomics score (rad-score)

**Fig. 4:**
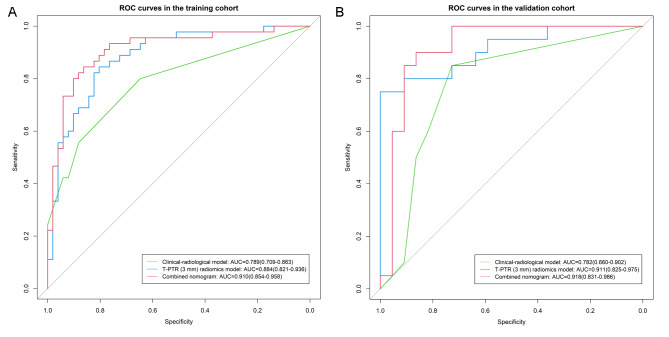
ROC curves for intratumoral and peritumoral region (T-PTR) (3 mm) radiomics model, clinical-radiological model, and combined nomogram in the training cohort (**A**) and the validation cohort (**B**)

The T-PTR (3 mm) rad-score, ALP, tumor size, and satellite nodule were considered as input variables for logistic regression to build the combined model. We chose a nomogram as the graphical representation of the best-performing combined model (shown in Fig. 3). The combined nomogram yielded an AUC, an accuracy, a sensitivity, and a specificity of 0.910 (95% CI, 0.854–0.958), 0.844, 0.822, and 0.863, respectively, for discriminating between the OR and NR groups in the training cohort and of 0.918 (95% CI, 0.831–0.986), 0.857, 0.800, and 0.909, respectively, in the validation cohort. The discriminating performance of the combined nomogram in the training cohort was significantly superior to that of the clinical-radiological model (AUC, 0.910 vs. 0.789, *P* = 0.028), whereas there was no significant difference between the models in the validation cohort (AUC, 0.918 vs. 0.782, *P* = 0.127). No significant differences were found between the T-PTR (3 mm) radiomics model and combined nomogram (training cohort, *P* = 0.577; validation cohort, *P* = 0.918) and between the T-PTR (3 mm) radiomics model and clinical-radiological model (training cohort, *P* = 0.094; validation cohort, *P* = 0.139). Table 2 summarizes the predictive performance of the constructed models. ROC curves for clinical-radiological model, T-PTR (3 mm) radiomics model, and combined nomogram in the both cohorts are shown in Fig. 4A and B. Delong’s test of different predictive models in the both cohorts is shown in Supplementary Data S9. Stratified analysis showed that each predictive model performed well in the two subgroups of inclusion time and the overall cohort (shown in Supplementary Data S10). The results of predictive models in the independent testing cohort are listed in Supplementary Data S6.

**Fig. 5:**
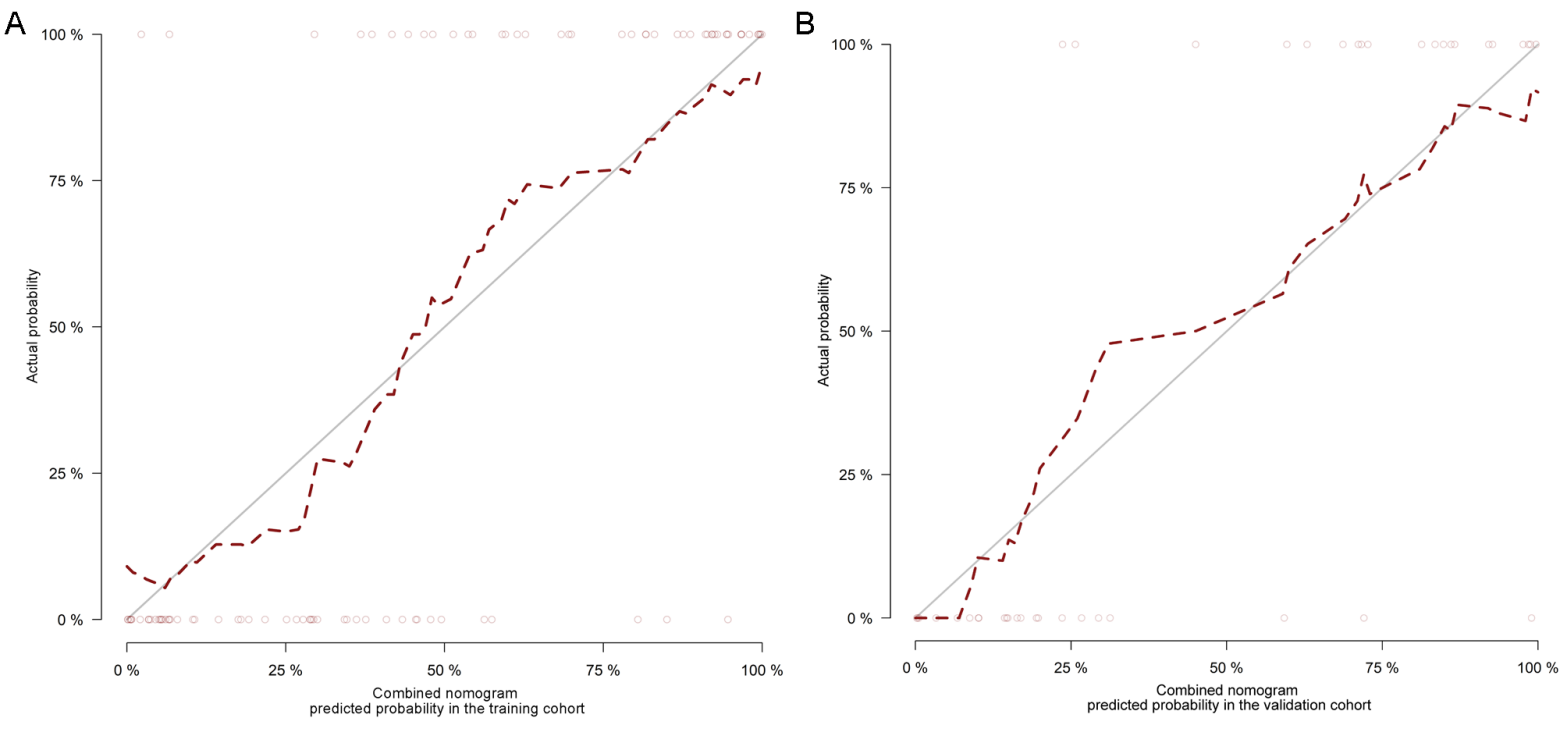
Calibration curves of the combined nomogram in the training cohort (**A**) and the validation cohort (**B**). The x-axis represents the predicted probability, and the y-axis represents the actual result. The diagonal solid line indicates the ideal prediction by a perfect model. The dashed line indicates the predictive performance of the model. If the dashed line is closer to the diagonal solid line, it means a better prediction

**Fig. 6:**
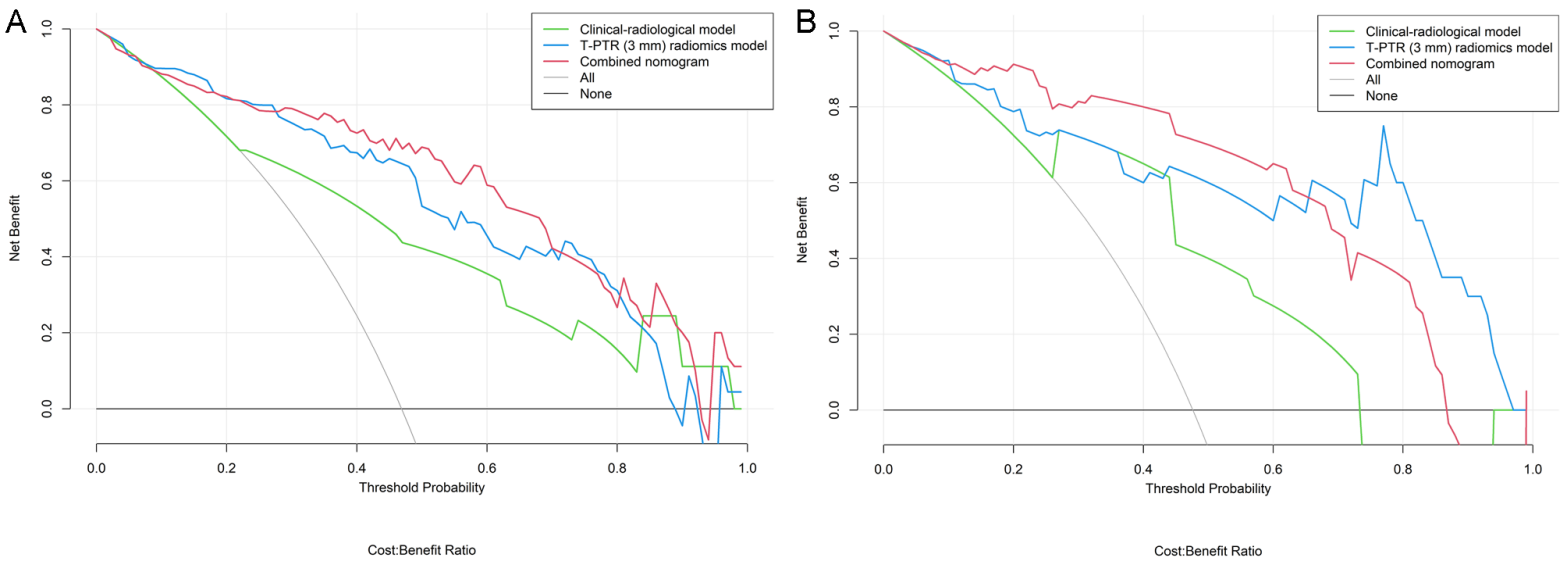
Decision curve analysis for the intratumoral and peritumoral region (T-PTR) (3 mm) radiomics model, clinical-radiological model, and combined nomogram in the training cohort (**A**) and the validation cohort (**B**). The y-axis represents the net benefit, and the x-axis represents the threshold probability. The (T-PTR) (3 mm) radiomics model, clinical-radiological model, and combined nomogram obtained more net benefits than either the treat-all-patients scheme or the treat-none-patients scheme for most of the threshold probabilities for predicting treatment response to TACE in patients with HCC.

Calibration curves of the combined nomogram for the probability of treatment response demonstrated good agreements between prediction and observation in the training and validation cohorts (Fig. 5A and B). The Hosmer-Lemeshow test yielded non-significant results in the both cohorts (*P* = 0.386 and 0.343), which suggested a satisfying fit of the nomogram. The DCA indicated that the combined nomogram obtained more net benefits than the clinical-radiological model, T-PTR (3 mm) radiomics model, and “treat-all” or “treat-none” strategies for most of the threshold probabilities in the training and validation cohorts (Fig. 6A and B).

## Discussion

In the study, we constructed various radiomics models of intratumoral, peritumoral, and intratumoral combined peritumoral derived from CE-MR images for preoperatively predicting treatment response of TACE in patients with HCC. Our study confirmed that intratumoral combined peritumoral radiomics models performed better than the intratumoral model. Furthermore, a combined nomogram incorporating clinical-radiological risk factors and the optimal T-PTR (3 mm) rad-score was developed and validated, and demonstrated satisfactory performance, calibration, and clinical utility. The proposed radiomics approach successfully predicted TACE treatment efficacy and may facilitate individualized treatment decision-making for patients with HCC.

In the present study, most of HCC patients receiving TACE had BCLC A or B stage, which was consistent with previous studies [18, 31]. The BCLC system recommends TACE as the standard therapy for intermediate HCC. TACE could be a candidate treatment option for early-stage patients who are unsuitable for resection or ablation due to old age, hepatic dysfunction, severe comorbidities, and tumor location [4]. This treatment stage-migration strategy is well established and recommended by international guidelines [28, 32]. Therefore, TACE clearly has a critical role in the treatment of HCC at early stage, and HCC patients included in our study reflect the real conditions in clinical setting.

Patients with HCC receiving TACE have various treatment efficacy and clinical outcomes [6, 7]. Objective response after first TACE course has been proved to be an independent and robust prognostic predictor for clinical outcomes, which may aid in guiding individual therapeutic strategies in HCC patients [33]. Several recent studies [34–37] have constructed radiomics models based on preoperative single MRI sequence or multiparametric MRI (MP-MRI) to predict tumor response of HCC patients receiving TACE, and the AUCs ranged from 0.692 to 0.866 in the validation cohort; however, their studies only focused on intratumoral radiomics features. Pathologically, peritumoral parenchyma is representative of cancerous heterogeneity, and the crucial information can be indicated by changes in the area surrounding tumors, such as biological aggressiveness, microinvasion, and micrometastasis [15, 18, 38]; thus, accurate evaluation of the neighboring tissue around tumors may also be useful in predicting treatment response and prognosis of TACE in patients with HCC.

Previous studies have reported that radiomics analysis of intratumoral combined 3 mm, 5 mm, and 10 mm peritumoral regions can provide valuable information for prognosis prediction in HCC [18, 19, 30, 39, 40]. In addition, according to the practice guidelines for the pathological diagnosis of primary liver cancer (2015 update) [41], the liver tissues within 10 mm surrounding the tumor are defined as the adjacent areas around the cancer, where the probability of MVI is high. Therefore, our study selected the most stable and predictive radiomics features from intratumoral, 3 mm, 5 mm, and 10 mm peritumoral regions for radiomics model construction, which can quantitatively assess the heterogeneity and invasiveness of intratumoral and peritumoral tissues in a non-invasive way. In the present study, the PTR (3 mm), PTR (5 mm), and PTR (10 mm) radiomics models showed comparable performance compared with the TR model, which indicated that peritumoral tissues were possess of a clinical value in assessing treatment efficacy. Yang et al. [42] reached a similar result that peritumoral radiomics model obtained equivalent performance compared with the intratumoral model in predicting MVI in HCC patients with the AUCs of 0.714 and 0.728, respectively. The radiomics features contributed to peritumoral model construction in the study were most derived from AP images. This finding was in agreement with previous studies, in which the presence of peritumoral enhancement in AP images indicated more aggressive biological behavior [18, 31].

We further combined intratumoral and peritumoral rad-scores to establish T-PTR (3 mm), T-PTR (5 mm), and T-PTR (10 mm) radiomics models for predicting TACE response. In our study, T-PTR radiomics models demonstrated better predictive performance compared with the TR radiomics model, which indicated that peritumoral radiomics might potentially enhance the ability of intratumoral radiomics for TACE response prediction. This might be interpreted that arterial peritumoral enhancement and irregular margin presented in the peritumoral area are independent predictors of prognosis in HCC patients [18, 19]. Chen et al. [30] found that intratumoral and peritumoral (5 mm, 10 mm, and 20 mm) radiomics models based on contrast-enhanced CT images performed better than the intratumoral radiomics model in predicting the first TACE response with the AUCs of 0.790, 0.810, 0.750, and 0.720, respectively, which was consistent with our study. Additionally, our study demonstrated that the T-PTR (3 mm) radiomics model achieved the best-performing performance among the seven radiomics models. A similar study reported by Liu et al. [40] found that intratumoral and peritumoral (3 mm) radiomics model showed better performance compared with radiomics models on intratumoral, peritumoral (3 mm), peritumoral (5 mm), and intratumoral and peritumoral (5 mm) for predicting 1-year survival of HCC after hepatectomy. Only one of the published studies, conducted MRI-based radiomics on intratumoral and peritumoral regions for TACE prognosis prediction [18]. In their study, radiomics models based on the entire tumor volumetric of AP (AP^ETV^), PVP^ETV^, and the border extensions of 1 mm, 3 mm, and 5 mm on the PVP (PVP^B1^, PVP^B3^, and PVP^B5^) were constructed to predict recurrence-free survival (RFS) of HCC patients after TACE. The best C-index results of PVP^ETV^ and PVP^B3^ radiomics models were 0.727 and 0.714 in the validation dataset, respectively. However, the above research only performed radiomics analysis of whole areas including intratumoral and peritumoral regions, and did not explore the individual contribution of the area around the tumor to predictive model construction; thus, it was unable to determine the significance of the separate peritumoral region in predicting recurrence or prognosis. Compared with the previous study [18], our study may have the following advantages: first, radiomics features derived from three-phase enhanced MR images might more fully reflect tumor heterogeneity and vascularization patterns, which is helpful for efficacy estimation; second, the individual peritumoral (3 mm, 5 mm, and 10 mm) radiomics models were constructed, and the valuable peritumoral distance was determined; third, intratumoral combined peritumoral radiomics analysis may contain more prognostic information, and potentially provide a more accurate and effective approach of individualized efficacy prediction for HCC patients.

In this study, during the construction of the clinical-radiological model, ALP, tumor size, and satellite nodule were independent predictors associated with treatment response of HCC after TACE. Previous researches on TACE clarified that a higher ALP value was an independent risk factor for unfavourable overall survival (OS) [43, 44]. Our study showed that abnormal ALP value was a significant predictor for poor response of HCC. Additionally, ALP has already been included in the Chinese University Prognostic Index, a HCC staging system that assigns a score of 3 when ALP is > 200 IU/L, indicating the potential role of ALP in predicting the prognosis of HCC patients [45]. Tumor size has been broadly recognized as a major predictive factor of treatment response for TACE [9, 33]. Larger tumors usually have more satellite lesions or daughter nodules making it difficult for TACE to achieve CR [46]. In our study, maximal tumor size > 5 cm was a significant predictive factor for NR, a result similar to the study by Jeong et al. [47]. Several studies reported that satellite nodule surrounding the main tumor was closely related to tumor grade, MVI, and early recurrence (ER) after resection, and TACE treatment efficacy and prognosis [10, 17, 37, 48]. Our study demonstrated that the presence of satellite nodule was inclined to show NR to TACE treatment. This may be interpreted that the development of satellite nodule favors vascular invasion and also tumor recurrence [48].

We ultimately developed a combined nomogram integrating the T-PTR (3 mm) rad-score with clinical-radiological risk indicators for treatment response prediction. The combined nomogram achieved good calibration and the strongest predictive performance based on AUCs in the training (nomogram vs. radiomics model vs. clinical-radiological model, 0.910 vs. 0.884 vs. 0.789) and validation (nomogram vs. radiomics model vs. clinical-radiological model, 0.918 vs. 0.911 vs. 0.782) cohorts. The novel combined nomogram was evaluated by a decision curve to clarify the clinical usefulness, which may offer insight into clinical outcomes on the basis of threshold probability, from which the net benefit could be derived [36]. Our results clearly demonstrated that the combined nomogram could obtain more net benefit than either the treat-all-patients or the treat-none-patients strategies across a wide range of threshold probabilities. Therefore, our novel nomogram may provide a reliable and efficient tool that enables visualized and personalized decision-making for the treatment management of patients with HCC.

This study has several limitations. Firstly, this was a retrospective study at a single center, which may introduce selection bias. The sample size was relatively small, especially for the independent testing cohort. A larger cohort population from multi-center is further needed to externally validate the robustness and reproducibility of the predictive models and reinforce the conclusions of our study. Secondly, the ROIs were manually delineated by radiologists, and thus is time-consuming and prone to error and user variability. It’s essential to develop an automatic and reliable liver tumor segmentation tool. Thirdly, it should be noted that MP-MRI data are not included in this study. In the future, we will attempt to develop a radiomics approach based on MP-MRI for response evaluation after TACE. Fourthly, for patients with multifocal HCCs, our study chose the largest lesion for radiomics analysis. A further direction will be considered to perform the per-lesion level study, as well as to explore how to comprehensively analyze radiomics features of multifocal lesions for treatment efficacy prediction. Finally, our study provided a promising tool for the precise prediction of treatment response after the initial TACE. In the future, we will try to explore MRI-based intratumoral and peritumoral radiomics features associated with RFS of HCC patients treated with TACE.

## Conclusions

In conclusion, intratumoral and peritumoral radiomics based on preoperative CE-MR images can enhance the ability of radiomics model in predicting tumor response to TACE. The combined nomogram which incorporated the rad-score and clinical-radiological risk factors provides an effective tool for the precise and individualized estimation of treatment response for HCC patients treated with TACE. The accurate identification of HCC patients who would receive benefit from upfront TACE might potentially help decision-making for subsequent treatment strategies.

### Electronic supplementary material

Below is the link to the electronic supplementary material.

Supplementary Material 1

## Data Availability

All data generated or analysed during this study are included in supplementary material of this article.

## Abbreviations

AASLD: American Association for the Study of Liver Disease
ADC: Apparent diffusion coefficient
AFP: Alpha-fetoprotein
ALB: Albumin
ALP: Alkaline phosphatase
ALT: Alanine aminotransferase
AP: Arterial phase
AST: Aspartate aminotransferase
AUC: Area under the curve
BCLC: Barcelona Clinic Liver Cancer
CE-MRI: Contrast-enhanced magnetic resonance imaging
CI: Confidence interval
CR: Complete response
DCA: Decision curve analysis
DP: Delayed phase
ECOG: Eastern Cooperative Oncology Group
EpCAM: Epithelial cell adhesion molecule
ER: Early recurrence
GBDT: Gradient boosting decision tree
GGT: γ-glutamyltranspeptadase
GLCM: Grey level co-occurrence matrix
GLRLM: Grey level run length matrix
GLZSM: Grey-level zone size matrix
HCC: Hepatocellular carcinoma
ICC: Intraclass correlation coefficient
MP-MRI: Multiparametric magnetic resonance imaging
mRECIST: Modified Response Evaluation Criteria in Solid Tumors
MVI: Microvascular invasion
NR: Non-response
OR: Objective response
OS: Overall survival
PD: Progression disease
PD-L1: Programmed death ligand 1
PLT: Platelet count
PR: Partial response
PT: Prothrombin time
PTR: Peritumoral region
PVP: Portal venous phase
Rad-score: Radiomics score
RFA: Radiofrequency ablation
RFS: Recurrence-free survival
ROC: Receiver operating characteristic curve
ROI: Region of interest
SD: Stable disease
TACE: Transarterial chemoembolization
TBIL: Total bilirubin
T-PTR: Intratumoral and peritumoral region
TR: Intratumoral region
VETC: Vessels encapsulating tumor clusters
VOI: Volume of interest

## References

[1] Sung H, Ferlay J, Siegel RL, Laversanne M, Soerjomataram I, Jemal A (2021). Global Cancer Statistics 2020: GLOBOCAN estimates of incidence and Mortality Worldwide for 36 cancers in 185 countries. CA Cancer J Clin.

[2] Vogel A, Meyer T, Sapisochin G, Salem R, Saborowski A (2022). Hepatocellular carcinoma. Lancet.

[3] Lin S, Hofmann K, Schemmer P (2012). Treatment of hepatocellular carcinoma: a systematic review. Liver Cancer.

[4] Chang Y, Jeong SW, Young Jang J, Jae Kim Y (2020). Recent updates of Transarterial Chemoembolilzation in Hepatocellular Carcinoma. Int J Mol Sci.

[5] Piscaglia F, Ogasawara S (2018). Patient selection for Transarterial Chemoembolization in Hepatocellular Carcinoma: Importance of Benefit/Risk Assessment. Liver Cancer.

[6] Guan YS, He Q, Wang MQ (2012). Transcatheter arterial chemoembolization: history for more than 30 years. ISRN Gastroenterol.

[7] Song YG, Shin SW, Cho SK, Choi D, Rhim H, Lee MW (2015). Transarterial chemoembolization as first-line therapy for hepatocellular carcinomas infeasible for ultrasound-guided radiofrequency ablation: a retrospective cohort study of 116 patients. Acta Radiol.

[8] Zhang H, He X, Yu J, Song W, Liu X, Liu Y (2019). Preoperative MRI features and clinical laboratory indicators for predicting the early therapeutic response of hepatocellular carcinoma to transcatheter arterial chemoembolization combined with high-intensity focused ultrasound treatment. Br J Radiol.

[9] Kim YJ, Lee MH, Choi SY, Yi BH, Lee HK (2019). Magnetic resonance imaging features predictive of an incomplete response to transarterial chemoembolization in patients with hepatocellular carcinoma: a STROBE-compliant study. Med (Baltim).

[10] Li ZW, Ren AH, Yang DW, Xu H, Wei J, Yuan CW (2022). Preoperatively predicting early response of HCC to TACE using clinical indicators and MRI features. BMC Med Imaging.

[11] Wu XM, Wang JF, Ji JS, Chen MG, Song JG (2017). Evaluation of efficacy of transcatheter arterial chemoembolization for hepatocellular carcinoma using magnetic resonance diffusion-weighted imaging. Onco Targets Ther.

[12] Aerts HJ (2016). The potential of radiomic-based phenotyping in precision medicine: a review. JAMA Oncol.

[13] Lambin P, Leijenaar RTH, Deist TM, Peerlings J, de Jong EEC, van Timmeren J (2017). Radiomics: the bridge between medical imaging and personalized medicine. Nat Rev Clin Oncol.

[14] Zhao Y, Chen R, Zhang T, Chen C, Muhelisa M, Huang J (2021). MRI-Based machine learning in differentiation between Benign and malignant breast lesions. Front Oncol.

[15] Tian Y, Hua H, Peng Q, Zhang Z, Wang X, Han J (2022). Preoperative evaluation of Gd-EOB-DTPA-Enhanced MRI Radiomics-Based Nomogram in Small Solitary Hepatocellular Carcinoma (≤ 3 cm) with Microvascular Invasion: a Two-Center Study. J Magn Reson Imaging.

[16] Shin J, Seo N, Baek SE, Son NH, Lim JS, Kim NK (2022). MRI Radiomics Model predicts pathologic complete response of rectal Cancer following Chemoradiotherapy. Radiology.

[17] Ji GW, Zhu FP, Xu Q, Wang K, Wu MY, Tang WW (2020). Radiomic features at contrast-enhanced CT predict recurrence in early stage Hepatocellular Carcinoma: a multi-institutional study. Radiology.

[18] Song W, Yu X, Guo D, Liu H, Tang Z, Liu X (2020). MRI-Based Radiomics: Associations with the recurrence-free survival of patients with Hepatocellular Carcinoma treated with conventional transcatheter arterial chemoembolization. J Magn Reson Imaging.

[19] Wang F, Cheng M, Du B, Li LM, Huang WP, Gao JB (2022). Use of radiomics containing an effective peritumoral area to predict early recurrence of solitary hepatocellular carcinoma ≤ 5 cm in diameter. Front Oncol.

[20] Zhu XD, Zhang JB, Zhuang PY, Zhu HG, Zhang W, Xiong YQ (2008). High expression of macrophage colony-stimulating factor in peritumoral liver tissue is associated with poor survival after curative resection of hepatocellular carcinoma. J Clin Oncol.

[21] Cheng Z, Yang P, Qu S, Zhou J, Yang J, Yang X (2015). Risk factors and management for early and late intrahepatic recurrence of solitary hepatocellular carcinoma after curative resection. HPB (Oxford).

[22] Dai XM, Huang T, Yang SL, Zheng XM, Chen GG, Zhang T (2017). Peritumoral EpCAM is an independent prognostic marker after curative resection of HBV-Related Hepatocellular Carcinoma. Dis Markers.

[23] Dai X, Xue J, Hu J, Yang SL, Chen GG, Lai PBS (2017). Positive expression of programmed death ligand 1 in Peritumoral Liver tissue is Associated with poor survival after curative resection of Hepatocellular Carcinoma. Transl Oncol.

[24] Kong LQ, Zhu XD, Xu HX, Zhang JB, Lu L, Wang WQ (2013). The clinical significance of the CD163 + and CD68 + Macrophages in patients with Hepatocellular Carcinoma. PLoS ONE.

[25] Xu X, Zhang HL, Liu QP, Sun SW, Zhang J, Zhu FP (2019). Radiomic analysis of contrast-enhanced CT predicts microvascular invasion and outcome in hepatocellular carcinoma. J Hepatol.

[26] Yu Y, Fan Y, Wang X, Zhu M, Hu M, Shi C (2022). Gd-EOB-DTPA-enhanced MRI radiomics to predict vessels encapsulating tumor clusters (VETC) and patient prognosis in hepatocellular carcinoma. Eur Radiol.

[27] Yuan G, Song Y, Li Q, Hu X, Zang M, Dai W (2021). Development and validation of a contrast-enhanced CT-Based Radiomics Nomogram for Prediction of Therapeutic Efficacy of Anti-PD-1 antibodies in Advanced HCC Patients. Front Immunol.

[28] Marrero JA, Kulik LM, Sirlin CB, Zhu AX, Finn RS, Abecassis MM (2018). Diagnosis, staging and management of hepatocellular carcinoma: 2018 Practice Guidance by the American Association for the study of Liver Diseases. Hepatology.

[29] Lencioni R, Llovet JM (2010). Modified RECIST (mRECIST) assessment for hepatocellular carcinoma. Semin Liver Dis.

[30] Chen M, Cao J, Hu J, Topatana W, Li S, Juengpanich S (2021). Clinical-radiomic analysis for pretreatment prediction of Objective Response to First Transarterial Chemoembolization in Hepatocellular Carcinoma. Liver Cancer.

[31] Meng XP, Wang YC, Ju S, Lu CQ, Zhong BY, Ni CF (2020). Radiomics Analysis on Multiphase contrast-enhanced CT: a Survival Prediction Tool in patients with Hepatocellular Carcinoma undergoing Transarterial Chemoembolization. Front Oncol.

[32] European Association for the Study of the Liver (2018). EASL Clinical Practice Guidelines: management of hepatocellular carcinoma. J Hepatol.

[33] Kim BK, Kim SU, Kim KA, Chung YE, Kim MJ, Park MS (2015). Complete response at first chemoembolization is still the most robust predictor for favorable outcome in hepatocellular carcinoma. J Hepatol.

[34] Sun Y, Bai H, Xia W, Wang D, Zhou B, Zhao X (2020). Predicting the Outcome of Transcatheter arterial embolization therapy for Unresectable Hepatocellular Carcinoma based on Radiomics of Preoperative Multiparameter MRI. J Magn Reson Imaging.

[35] Kuang Y, Li R, Jia P, Ye W, Zhou R, Zhu R (2021). MRI-Based Radiomics: Nomograms predicting the short-term response after transcatheter arterial chemoembolization (TACE) in hepatocellular carcinoma patients with diameter less than 5 cm. Abdom Radiol (NY).

[36] Kong C, Zhao Z, Chen W, Lv X, Shu G, Ye M (2021). Prediction of tumor response via a pretreatment MRI radiomics-based nomogram in HCC treated with TACE. Eur Radiol.

[37] Liu QP, Yang KL, Xu X, Liu XS, Qu JR, Zhang YD (2022). Radiomics analysis of pretreatment MRI in predicting tumor response and outcome in hepatocellular carcinoma with transarterial chemoembolization: a two-center collaborative study. Abdom Radiol (NY).

[38] Budhu A, Forgues M, Ye QH, Jia HL, He P, Zanetti KA (2006). Prediction of venous metastases, recurrence, and prognosis in hepatocellular carcinoma based on a unique immune response signature of the liver microenvironment. Cancer Cell.

[39] Li N, Wan X, Zhang H, Zhang Z, Guo Y, Hong D (2022). Tumor and peritumor radiomics analysis based on contrast-enhanced CT for predicting early and late recurrence of hepatocellular carcinoma after liver resection. BMC Cancer.

[40] Liu Y, Wei X, Zhang X, Pang C, Xia M, Du Y (2022). CT radiomics combined with clinical variables for predicting the overall survival of hepatocellular carcinoma patients after hepatectomy. Transl Oncol.

[41] Cong WM, Bu H, Chen J, Dong H, Zhu YY, Feng LH (2016). Guideline Committee. Practice guidelines for the pathological diagnosis of primary liver cancer: 2015 update. World J Gastroenterol.

[42] Yang Y, Fan W, Gu T, Yu L, Chen H, Lv Y (2021). Radiomic features of Multi-ROI and multi-phase MRI for the prediction of Microvascular Invasion in Solitary Hepatocellular Carcinoma. Front Oncol.

[43] Le Y, Shen JX, Zhang YF, He MK, Kan A, Chen HL (2019). Transarterial Chemoembolization related to good survival for selected patients with advanced Hepatocellular Carcinoma. J Cancer.

[44] Wang H, Du PC, Wu MC, Cong WM (2018). Postoperative adjuvant transarterial chemoembolization for multinodular hepatocellular carcinoma within the Barcelona Clinic Liver Cancer early stage and microvascular invasion. Hepatobiliary Surg Nutr.

[45] Leung TW, Tang AM, Zee B, Lau WY, Lai PB, Leung KL (2002). Construction of the Chinese University Prognostic Index for hepatocellular carcinoma and comparison with the TNM staging system, the Okuda staging system, and the Cancer of the liver italian program staging system: a study based on 926 patients. Cancer.

[46] Bannangkoon K, Hongsakul K, Tubtawee T, McNeil E, Sriplung H, Chongsuvivatwong V (2018). Rate and predictive factors for sustained complete response after selective Transarterial chemoembolization (TACE) in patients with Hepatocellular Carcinoma. Asian Pac J Cancer Prev.

[47] Jeong SO, Kim EB, Jeong SW, Jang JY, Lee SH, Kim SG (2017). Predictive factors for complete response and recurrence after Transarterial Chemoembolization in Hepatocellular Carcinoma. Gut Liver.

[48] Ahn SJ, Kim JH, Park SJ, Kim ST, Han JK (2019). Hepatocellular carcinoma: preoperative gadoxetic acid-enhanced MR imaging can predict early recurrence after curative resection using image features and texture analysis. Abdom Radiol (NY).

