# Supplementary material for: Intratumoral and peritumoral radiomics based on contrast-enhanced MRI for preoperatively predicting treatment response of transarterial chemoembolization in hepatocellular carcinoma
Source: BMC Cancer. 2023 Oct 24;23:1026. doi: 10.1186/s12885-023-11491-0 (PMC10594790; doi:10.1186/s12885-023-11491-0)
Supplement: Supplementary file 1 — Supplementary Material 1 [file 12885_2023_11491_MOESM1_ESM.doc]

**Supplementary Data**

**S1. Magnetic resonance imaging sequences and parameters**

| Acquisition sequences | Field strength | Echo time (ms) | Repetition time (ms) | FOV (cm^2^) | Matrix size | NEX | Section thickness (mm) | Intersection gap (mm) |
| --- | --- | --- | --- | --- | --- | --- | --- | --- |
| Dual-echo T1-weighted gradient-recalled-echo | 1.5 T | 4.2 and 2.1 | 5.8 | 40 × 36 | 256 × 170 | 0.70 | 5 | -2.5 |
|  | 3.0 T | 2.5 and 1.2 | 4 | 40 × 36 | 288 × 224 | 0.69 | 5 | -2.5 |
| T2-weighted spin-echo | 1.5 T | 92.6 | 6667 | 44 × 33 | 256 × 256 | 2.00 | 6.5 | 1.5 |
|  | 3.0 T | 96.1 | 7059 | 44 × 39.6 | 288 × 224 | 2.00 | 6 | 1 |
| Contrast-enhanced imaging | 1.5 T | 3.1 | 5.8 | 40 × 36 | 256 × 170 | 0.70 | 5 | -2.5 |
|  | 3.0 T | 1.8 | 4 | 42 × 37.8 | 270 × 224 | 0.70 | 5 | -2.5 |

FOV, field of view; NEX, number of excitation.

**S2. Detailed definitions of imaging features and representative MR images**

The radiologists evaluated the following imaging traits for each patient: (1) tumor size (the maximum axial diameter including the capsule measured on portal venous phase (PVP) images); (2) tumor number (unifocal or multifocal); (3) tumor margin (smooth or non-smooth); (4) intratumoral necrosis (defined as nonenhancing areas with an intensity similar to that of gallbladder contents); (5) intratumoral hemorrhage (defined as heterogeneous hyperintensity on T1WI and hypointensity on T2WI); (6) intratumoral fat (defined as signal intensity drop on opposed-phase T1W images compared to in-phase images); (7) tumor encapsulation (defined as a peripheral rim of hyperenhancement on PVP or delayed phase (DP) images); (8) arterial peritumoral enhancement (defined as detectable enhancing portions adjacent to the tumor border on arterial phase (AP) images, later becoming isointensity compared with the background liver parenchyma on DP images); (9) satellite nodule (defined as small tumor nodule that was separated from the primary tumor with a similar type of enhancement, and had a tumor diameter and distance from the main tumor of no more than 2 cm); (10) internal arteries (defined as the discrete arteries within the tumor); (11) radiological cirrhosis (defined as surface irregularity and nodularity, shrunken of liver, accompanied by ascites or signs of portal hypertension).

Representative MR images of major imaging features. (a) Tumor size was the maximum diameter including the capsule on portal venous phase (PVP) images. (b) Non-smooth margin presented as nonnodular tumor with irregular contour. (c) Tumor encapsulation was a peripheral rim of hyperenhancement on PVP images. (d) Arterial peritumoral enhancement was a zone of hyperenhancement around the tumor on arterial phase (AP) images. (e) Satellite nodule was a lesion size less than 2 cm, with a similar type of enhancement, and a distance within 2 cm from the main tumor. (f) Internal arteries showed discrete arteries within the tumor.

**S3. Detailed description of TACE procedure**

All conventional TACE procedures were carried out by interventional radiologists with no less than 10 years of clinical experience. The Seldinger technique was used to puncture the right femoral artery, and a 5F catheter (RH catheter, Terumo, Tokyo, Japan) was inserted for celiac and hepatic artery angiography in evaluation of the vascular anatomy and tumor burden. For HCC patients who received TACE during April 2008 and June 2015, after superselective catheterization of hepatic artery feeding the tumor, an emulsion mixture of doxorubicin, hydroxycamptothecin, and Lipiodol was injected using a 2.7F microcatheter, followed by embolization of the tumor-feeding artery using gelatin sponge particles (1-2 mm). For HCC patients who received TACE during July 2015 and February 2022, after superselective catheterization of hepatic artery feeding the tumor, an emulsion mixture of lobaplatin, raltitrexed, and Lipiodol was injected using a 2.7F microcatheter, followed by embolization of the tumor-feeding artery using blank microsphere. TACE procedure was stopped when tumor staining completely disappeared and regional arterial blood flow stopped.

Between April 2008 and June 2015, the patients for OR and NR were 31 (48.4%) and 33 (51.6%), respectively. Between July 2015 and February 2022, the patients for OR and NR were 42 (56.8%) and 32 (43.2%), respectively. The OR rate between the two subgroups of different inclusion time was not significantly different (48.4% vs. 56.8%, *F* = 1.624, *P* = 0.203).

**S4. Visualization of tumor response assessment**

The target hepatocellular carcinoma (HCC) lesion at baseline (a) showed an enhancing tumor diameter of 4.34 cm, while post-treatment (b) enhancing tumor diameter was 2.16 cm. Decrease of viable portion with a variation rate of 50.23 % in the arterial phase, demonstrating partial response according to the mRECIST criterion (defined as objective response). The target HCC lesion at baseline (c) showed an enhancing tumor diameter of 6.47 cm, while post-treatment (d) enhancing tumor diameter was 5.87 cm. Decrease of viable portion with a variation rate of 9.27 % in the arterial phase, demonstrating stable disease (defined as non-response).

**S5. Details of radiomics features**

**5.1 Histogram features**

Histogram parameters are concerned with properties of individual pixels. They describe the distribution of voxel intensities within the MR image through commonly used and basic metrics. Let $X$ denote the three dimensional image matrix with $N$ voxels, and let $P$ denote the first order histogram divided by $N_{l}$ discrete intensity levels.

**1) MinIntensity:** The minimum intensity value of $X$.

**2) MaxIntensity:** The maximum intensity value of $X$.

**3) MedianIntensity:** The median intensity value of $X$.

## **4) Mean:**

5) **StandardDeviation:**

Where $\overline{X}$ is the mean of $X$.

## **6) MeanDeviation:** The mean of the absolute deviations of all voxel intensities around the mean intensity value.

## **7) RelativeDeviation:** Let $\overline{X}$ denote the mean of a set of quantities $X_{i}$, then the relative deviation is defined by:

## **8) Variance:**

Where $\overline{X}$ is the mean of $X$.

## **9) Range:** The range of intensity values of $X$.

## **10) VolumeCount:** Describe the size of the ROI.

## **11) VoxelValueSum:** Represents the Sum calculations for voxels in the ROI.

## **12) RootMeanSquare (RMS):**

## **13) Skewness:**

Where $\overline{X}$ is the mean of $X$.

**14) Kurtosis:**

Where $\overline{X}$ is the mean of $X$.

## **15) Uniformity:**

## **16) Energy:**

**17) Entropy:**

## **18) FrequencySize:** The ratio of the frequency of each object to the total frequency.

## **19) Percentile:** The percentile (p%) is defined as that value of the brightness $a$:

or equivalently:

The P-th percentile of a list of N ordered values (sorted from least to greatest) is the smallest value in the list such that P percent of the data is less than or equal to that value. This is obtained by first calculating the ordinal rank and then taking the value from the ordered list that corresponds to that rank. The ordinal rank n is calculated using this formula:

The 19 Percentiles are extracted: Percentile5, Percentile10, Percentile15, Percentile20, Percentile25, Percentile30, Percentile35, Percentile40, Percentile45, Percentile50, Percentile55, Percentile60, Percentile65, Percentile70, Percentile75, Percentile80, Percentile85, Percentile90, Percentile95.

**20) Quantile:** For a finite population of *N* equally probable values indexed 1, …, *N* from lowest to highest, the k-th quantile of this population can equivalently be computed via the value of:

The 5 Quantiles are extracted: Quantile0.025, Quantile0.25, Quantile0.5, Quantile0.75, Quantile0.975.

**5.2 Form factors**

These group of features includes descriptors of the three-dimensional size and shape of the tumor region. Let in the following definitions *V* denote the volume and *A* the surface area of the volume of interest. We determined the following shape and size based features:

**1) MeshVolume:**

Formula:

The volume of the ROI *V* is calculated from the triangle mesh of the ROI. For each face *i* in the mesh, defined by points *a_i_*, *b_i_* and *c_i_*, the (signed) volume *V_f_* of the tetrahedron defined by that face and the origin of the image (*O*) is calculated.

**2) OneVoxelVolume:** The volume of a single voxel.

**3) VoxelVolume:** The volume (*V*) of the tumor is determined by counting the number of pixels in the tumor region and multiplying this value by the voxel size.

**4) Surface Area:**

**5) Surface to Volume Ratio:**

**6) Sphericity:**

**7) Compactness 1:**

**8) Compactness 2:**

**9) Spherical Disproportion:**

Where *R* is the radius of a sphere with the same volume as the tumor.

Where *N* is the total number of triangles covering the surface and *a*, *b* and *c* are edge vectors of the triangles.

**10) Maximum 3D diameter:** The maximum three-dimensional tumor diameter is measured as the largest pairwise Euclidean distance, between voxels on the surface of the tumor volume.

**11) MajorAxisLength:**

This feature yield the largest axis length of the ROI-enclosing ellipsoid and is calculated using the largest principal component *λ_major_*.

**12) MinorAxisLength:**

This feature yield the second-largest axis length of the ROI-enclosing ellipsoid and is calculated using the largest principal component *λ_minor_*.

**13) LeastAxisLength:**

This feature yield the smallest axis length of the ROI-enclosing ellipsoid and is calculated using the largest principal component *λ_least_*.

**14) Elongation:**

Here, *λ_major_* and *λ_minor_* are the lengths of the largest and second largest principal component axes.

**15) Flatness:**

Here, *λ_major_* and *λ_least_* are the lengths of the largest and smallest principal component axes. The values range between 1 (non-flat, sphere-like) and 0 (a flat object, or single-slice segmentation).

**5.3 Haralick features**

### 1) HaraEntropy:

Formula:

where $\mu_{t}$ and $\sigma_{t}$ are the mean and standard deviation of the row (or column, due to symmetry) sums.

### 2) Angular Second Moment:

Formula:

### 3) Contrast:

Formula:

### 4) Haralick Variance:

Formula:

### 5) SumAverage:

Formula:

### 6) SumVariance:

Formula:

### 7) SumEntropy:

Formula:

### 8) Difference Variance:

Formula:

### 9) Difference Entropy:

Formula:

### 10) Inverse Difference Moment:

Formula:

**5.4 Grey level co-occurrence matrix (GLCM) features**

The grey level co-occurrence matrix (GLCM) represents the joint probability of certain sets of pixels having certain grey-level values. It calculates how many times a pixel with grey-level i occurs jointly with another pixel having a grey value j. By varying the displacement vector d between each pair of pixels.

The rotation angle of an offset: 0°, 45°, 90°, 135° and displacement vectors (distance to the neighbor pixel: 1, 2, 3 ...), different co-occurrence distributions from the same image of reference. GLCM of an image is computed using displacement vector d defined by its radius, (distance or count to the next adjacent neighbor preferably is equal to one) and rotational angles.

### 1) GLCMEnergy:

### Formula:

g is a GLCM

Where i,j are the spatial coordinates of g (i,j).

*Angle=All Direction, 0, 45, 90, 135, All_SD;

*Offset=1, 4, 7

The 18 features of GLCMEnergy are extracted:

GLCMEnergy_AllDirection_offset1, GLCMEnergy_AllDirection_offset1_SD, GLCMEnergy_angle0_offset1, GLCMEnergy_angle45_offset1,

GLCMEnergy_angle90_offset1, GLCMEnergy_angle135_offset1,

GLCMEnergy_AllDirection_offset4, GLCMEnergy_AllDirection_offset4_SD, GLCMEnergy_angle0_offset4, GLCMEnergy_angle45_offset4,

GLCMEnergy_angle90_offset4, GLCMEnergy_angle135_offset4,

GLCMEnergy_AllDirection_offset7, GLCMEnergy_angle0_offset7, GLCMEnergy_angle45_offset7, GLCMEnergy_angle90_offset7, GLCMEnergy_angle135_offset7, GLCMEnergy_AllDirection_offset7_SD

### 2) GLCMEntropy:

Formula:

*Angle=All Direction, 0, 45, 90, 135, All_SD;

*Offset=1, 4, 7

The 18 features of GLCMEntropy are extracted:

GLCMEntropy_AllDirection_offset1, GLCMEntropy_AllDirection_offset1_SD, GLCMEntropy_angle0_offset1, GLCMEntropy_angle45_offset1, GLCMEntropy_angle90_offset1, GLCMEntropy_angle135_offset1,

GLCMEntropy_AllDirection_offset4, GLCMEntropy_AllDirection_offset4_SD, GLCMEntropy_angle0_offset4, GLCMEntropy_angle45_offset4, GLCMEntropy_angle90_offset4, GLCMEntropy_angle135_offset4,

GLCMEntropy_AllDirection_offset7, GLCMEntropy_AllDirection_offset7_SD, GLCMEntropy_angle0_offset7, GLCMEntropy_angle45_offset7, GLCMEntropy_angle90_offset7, GLCMEntropy_angle135_offset7

### 3) GLCMInertia:

Formula:

*Angle=All Direction, 0, 45, 90, 135, All_SD;

*Offset=1, 4, 7

The 18 features of GLCMInertia are extracted:

Inertia_AllDirection_offset1, Inertia _AllDirection_offset1_SD,

Inertia _angle0_offset1, Inertia _angle45_offset1,

Inertia _angle90_offset1, Inertia _angle135_offset1,

Inertia _AllDirection_offset4, Inertia _AllDirection_offset4_SD,

Inertia _angle0_offset4, Inertia _angle45_offset4,

Inertia _angle90_offset4, Inertia _angle135_offset4,

Inertia _AllDirection_offset7, Inertia _AllDirection_offset7_SD,

Inertia _angle0_offset7, Inertia _angle45_offset7,

Inertia _angle90_offset7, Inertia _angle135_offset7

### 4) Correlation:

Formula:

*Angle=All Direction, 0, 45, 90, 135, All_SD;

*Offset=1, 4, 7

The 18 features of Correlation are extracted:

Correlation_AllDirection_offset1, Correlation _AllDirection_offset1_SD,

Correlation _angle0_offset1, Correlation _angle45_offset1,

Correlation _angle90_offset1, Correlation _angle135_offset1,

Correlation _AllDirection_offset4, Correlation _AllDirection_offset4_SD,

Correlation _angle0_offset4, Correlation _angle45_offset4,

Correlation _angle90_offset4, Correlation _angle135_offset4,

Correlation _AllDirection_offset7, Correlation _AllDirection_offset7_SD,

Correlation _angle0_offset7, Correlation _angle45_offset7,

Correlation _angle90_offset7, Correlation _angle135_offset7

### 5) InverseDifferenceMoment:

Formula:

*Angle=All Direction, 0, 45, 90, 135, All_SD;

*Offset=1, 4, 7

The 18 features of InverseDifferenceMoment are extracted:

InverseDifferenceMoment_AllDirection_offset1, InverseDifferenceMoment_AllDirection_offset1_SD,

InverseDifferenceMoment_angle0_offset1,

InverseDifferenceMoment_angle45_offset1,

InverseDifferenceMoment_angle90_offset1,

InverseDifferenceMoment_angle135_offset1,

InverseDifferenceMoment_AllDirection_offset4,

InverseDifferenceMoment_AllDirection_offset4_SD,

InverseDifferenceMoment_angle0_offset4,

InverseDifferenceMoment_angle45_offset4,

InverseDifferenceMoment_angle90_offset4,

InverseDifferenceMoment_angle135_offset4,

InverseDifferenceMoment_AllDirection_offset7,

InverseDifferenceMoment_AllDirection_offset7_SD,

InverseDifferenceMoment_angle0_offset7,

InverseDifferenceMoment_angle45_offset7,

InverseDifferenceMoment_angle90_offset7,

InverseDifferenceMoment_angle135_offset7

### 6) ClusterShade:

Formula:

*Angle=All Direction, 0, 45, 90, 135, All_SD;

*Offset=1, 4, 7

The 18 features of ClusterShade are extracted:

ClusterShade_AllDirection_offset1, ClusterShade_AllDirection_offset1_SD,

ClusterShade_angle0_offset1, ClusterShade_angle45_offset1,

ClusterShade_angle90_offset1, ClusterShade_angle135_offset1,

ClusterShade_AllDirection_offset4, ClusterShade_AllDirection_offset4_SD,

ClusterShade_angle0_offset4, ClusterShade_angle45_offset4,

ClusterShade_angle90_offset4, ClusterShade_angle135_offset4,

ClusterShade_AllDirection_offset7, ClusterShade_AllDirection_offset7_SD,

ClusterShade_angle0_offset7, ClusterShade_angle45_offset7,

ClusterShade_angle90_offset7, ClusterShade_angle135_offset7

### 7) ClusterProminence:

Formula:

*Angle=All Direction, 0, 45, 90, 135, All_SD;

*Offset=1, 4, 7

The 18 features of ClusterProminence are extracted:

ClusterProminence_AllDirection_offset1, ClusterProminence_AllDirection_offset1_SD,

ClusterProminence_angle0_offset1, ClusterProminence_angle45_offset1,

ClusterProminence_angle90_offset1, ClusterProminence_angle135_offset1,

ClusterProminence_AllDirection_offset4, ClusterProminence_AllDirection_offset4_SD,

ClusterProminence_angle0_offset4, ClusterProminence_angle45_offset4,

ClusterProminence_angle90_offset4, ClusterProminence_angle135_offset4,

ClusterProminence_AllDirection_offset7, ClusterProminence_AllDirection_offset7_SD,

ClusterProminence_angle0_offset7, ClusterProminence_angle45_offset7,

ClusterProminence_angle90_offset7, ClusterProminence_angle135_offset7

### 8) HaralickCorrelation:

Formula:

where $\mu_{t}$ and $\sigma_{t}$ are the mean and standard deviation of the row (or column, due to

symmetry) sums.

*Angle=All Direction, 0, 45, 90, 135, All_SD;

*Offset=1, 4, 7

The 18 features of HaralickCorrelation are extracted:

HaralickCorrelation_AllDirection_offset1, HaralickCorrelation_AllDirection_offset1_SD,

HaralickCorrelation_angle0_offset1, HaralickCorrelation_angle45_offset1,

HaralickCorrelation_angle90_offset1, HaralickCorrelation_angle135_offset1,

HaralickCorrelation_AllDirection_offset4, HaralickCorrelation_AllDirection_offset4_SD,

HaralickCorrelation_angle0_offset4, HaralickCorrelation_angle45_offset4,

HaralickCorrelation_angle90_offset4, HaralickCorrelation_angle135_offset4,

HaralickCorrelation_AllDirection_offset7, HaralickCorrelation_AllDirection_offset7_SD,

HaralickCorrelation_angle0_offset7, HaralickCorrelation_angle45_offset7,

HaralickCorrelation_angle90_offset7, HaralickCorrelation_angle135_offset7

**5.5 Grey level run length matrix (GLRLM) features**

The grey level run-length matrix (RLM) **P_r_ (i**, **j | θ**) is defined as the numbers of runs with pixels of grey level *i* and run length *j* for a given direction θ. RLMs is generated for each sample image segment having directions (0°,45°,90° &135°), then the following ten statistical features were derived.

1) **ShortRunEmphasis：**

$$SRE(\theta)=\frac{1}{n_{r}}\sum_{i=1}^{M} \sum_{j=1}^{N} \frac{p(i,j,\theta)}{j^{2}}$$

*Angle=All Direction, 0, 45, 90, 135, All_SD;

*Offset=1, 4, 7

The 18 features of ShortRunEmphasis are extracted:

ShortRunEmphasis_AllDirection_offset1, ShortRunEmphasis_AllDirection_offset1_SD,

ShortRunEmphasis_angle0_offset1, ShortRunEmphasis_angle45_offset1,

ShortRunEmphasis_angle90_offset1, ShortRunEmphasis_angle135_offset1,

ShortRunEmphasis_AllDirection_offset4, ShortRunEmphasis_AllDirection_offset4_SD,

ShortRunEmphasis_angle0_offset4, ShortRunEmphasis_angle45_offset4,

ShortRunEmphasis_angle90_offset4, ShortRunEmphasis_angle135_offset4,

ShortRunEmphasis_AllDirection_offset7, ShortRunEmphasis_AllDirection_offset7_SD,

ShortRunEmphasis_angle0_offset7, ShortRunEmphasis_angle45_offset7,

ShortRunEmphasis_angle90_offset7, ShortRunEmphasis_angle135_offset7

2) **LongRunEmphasis：**

$$LRE(\theta)=\frac{1}{n_{r}}\sum_{i=1}^{M} \sum_{j=1}^{N} p(i,j,\theta)j^{2}$$

*Angle=All Direction, 0, 45, 90, 135, All_SD;

*Offset=1, 4, 7

The 18 features of LongRunEmphasis are extracted:

LongRunEmphasis_AllDirection_offset1, LongRunEmphasis_AllDirection_offset1_SD,

LongRunEmphasis_angle0_offset1, LongRunEmphasis_angle45_offset1,

LongRunEmphasis_angle90_offset1, LongRunEmphasis_angle135_offset1,

LongRunEmphasis_AllDirection_offset4, LongRunEmphasis_AllDirection_offset4_SD,

LongRunEmphasis_angle0_offset4, LongRunEmphasis_angle45_offset4,

LongRunEmphasis_angle90_offset4, LongRunEmphasis_angle135_offset4,

LongRunEmphasis_AllDirection_offset7, LongRunEmphasis_AllDirection_offset7_SD,

LongRunEmphasis_angle0_offset7, LongRunEmphasis_angle45_offset7,

LongRunEmphasis_angle90_offset7, LongRunEmphasis_angle135_offset7

3) **GreyLevelNonuniformity：**

$$GLN(\theta)=\frac{1}{n_{r}}\sum_{i=1}^{M} (\sum_{j=1}^{N} p(i,j,\theta))^{2}$$

*Angle=All Direction, 0, 45, 90, 135, All_SD;

*Offset=1, 4, 7

The 18 features of GreyLevelNonuniformity are extracted:

GreyLevelNonuniformity_AllDirection_offset1, GreyLevelNonuniformity_AllDirection_offset1_SD,

GreyLevelNonuniformity_angle0_offset1, GreyLevelNonuniformity_angle45_offset1,

GreyLevelNonuniformity_angle90_offset1, GreyLevelNonuniformity_angle135_offset1,

GreyLevelNonuniformity_AllDirection_offset4, GreyLevelNonuniformity_AllDirection_offset4_SD,

GreyLevelNonuniformity_angle0_offset4, GreyLevelNonuniformity_angle45_offset4,

GreyLevelNonuniformity_angle90_offset4, GreyLevelNonuniformity_angle135_offset4,

GreyLevelNonuniformity_AllDirection_offset7, GreyLevelNonuniformity_AllDirection_offset7_SD,

GreyLevelNonuniformity_angle0_offset7, GreyLevelNonuniformity_angle45_offset7,

GreyLevelNonuniformity_angle90_offset7, GreyLevelNonuniformity_angle135_offset7

4) **RunLengthNonuniformity：**

$$RLN(\theta)=\frac{1}{n_{r}}\sum_{j=i}^{N} (\sum_{i=1}^{M} p(i,j,\theta))^{2}$$

*Angle=All Direction, 0, 45, 90, 135, All_SD;

*Offset=1, 4, 7

The 18 features of RunLengthNonuniformity are extracted:

RunLengthNonuniformity_AllDirection_offset1, RunLengthNonuniformity_AllDirection_offset1_SD,

RunLengthNonuniformity_angle0_offset1, RunLengthNonuniformity_angle45_offset1,

RunLengthNonuniformity_angle90_offset1, RunLengthNonuniformity_angle135_offset1,

RunLengthNonuniformity_AllDirection_offset4, RunLengthNonuniformity_AllDirection_offset4_SD,

RunLengthNonuniformity_angle0_offset4, RunLengthNonuniformity_angle45_offset4,

RunLengthNonuniformity_angle90_offset4, RunLengthNonuniformity_angle135_offset4,

RunLengthNonuniformity_AllDirection_offset7, RunLengthNonuniformity_AllDirection_offset7_SD,

RunLengthNonuniformity_angle0_offset7, RunLengthNonuniformity_angle45_offset7,

RunLengthNonuniformity_angle90_offset7, RunLengthNonuniformity_angle135_offset7

5) **LowGreyLevelRunEmphasis：**

$$LGRE(\theta)=\frac{1}{n_{r}}\sum_{j=i}^{N} \sum_{i=1}^{M} \frac{p(i,j,\theta)}{i^{2}}$$

*Angle=All Direction, 0, 45, 90, 135, All_SD;

*Offset=1, 4, 7

The 18 features of LowGreyLevelRunEmphasis are extracted:

LowGreyLevelRunEmphasis_AllDirection_offset1, LowGreyLevelRunEmphasis_AllDirection_offset1_SD,

LowGreyLevelRunEmphasis_angle0_offset1, LowGreyLevelRunEmphasis_angle45_offset1,

LowGreyLevelRunEmphasis_angle90_offset1, LowGreyLevelRunEmphasis_angle135_offset1,

LowGreyLevelRunEmphasis_AllDirection_offset4, LowGreyLevelRunEmphasis_AllDirection_offset4_SD,

LowGreyLevelRunEmphasis_angle0_offset4, LowGreyLevelRunEmphasis_angle45_offset4,

LowGreyLevelRunEmphasis_angle90_offset4, LowGreyLevelRunEmphasis_angle135_offset4,

LowGreyLevelRunEmphasis_AllDirection_offset7, LowGreyLevelRunEmphasis_AllDirection_offset7_SD,

LowGreyLevelRunEmphasis_angle0_offset7, LowGreyLevelRunEmphasis_angle45_offset7,

LowGreyLevelRunEmphasis_angle90_offset7, LowGreyLevelRunEmphasis_angle135_offset7

6) **HighGreyLevelRunEmphasis：**

$$HGRE(\theta)=\frac{1}{n_{r}}\sum_{j=i}^{N} \sum_{i=1}^{M} p(i,j,\theta)i^{2}$$

*Angle=All Direction, 0, 45, 90, 135, All_SD;

*Offset=1, 4, 7

The 18 features of HighGreyLevelRunEmphasis are extracted:

HighGreyLevelRunEmphasis_AllDirection_offset1, HighGreyLevelRunEmphasis_AllDirection_offset1_SD,

HighGreyLevelRunEmphasis_angle0_offset1, HighGreyLevelRunEmphasis_angle45_offset1,

HighGreyLevelRunEmphasis_angle90_offset1, HighGreyLevelRunEmphasis_angle135_offset1,

HighGreyLevelRunEmphasis_AllDirection_offset4, HighGreyLevelRunEmphasis_AllDirection_offset4_SD,

HighGreyLevelRunEmphasis_angle0_offset4, HighGreyLevelRunEmphasis_angle45_offset4,

HighGreyLevelRunEmphasis_angle90_offset4, HighGreyLevelRunEmphasis_angle135_offset4,

HighGreyLevelRunEmphasis_AllDirection_offset7, HighGreyLevelRunEmphasis_AllDirection_offset7_SD,

HighGreyLevelRunEmphasis_angle0_offset7, HighGreyLevelRunEmphasis_angle45_offset7,

HighGreyLevelRunEmphasis_angle90_offset7, HighGreyLevelRunEmphasis_angle135_offset7

7) **ShortRunLowGreyLevelEmphasis：**

$$SRLGE(\theta)=\frac{1}{n_{r}}\sum_{j=i}^{N} \sum_{i=1}^{M} \frac{p(i,j,\theta)}{i^{2}j^{2}}$$

*Angle=All Direction, 0, 45, 90, 135, All_SD;

*Offset=1, 4, 7

The 18 features of ShortRunLowGreyLevelEmphasis are extracted:

ShortRunLowGreyLevelEmphasis_AllDirection_offset1, ShortRunLowGreyLevelEmphasis_AllDirection_offset1_SD,

ShortRunLowGreyLevelEmphasis_angle0_offset1, ShortRunLowGreyLevelEmphasis_angle45_offset1,

ShortRunLowGreyLevelEmphasis_angle90_offset1, ShortRunLowGreyLevelEmphasis_angle135_offset1,

ShortRunLowGreyLevelEmphasis_AllDirection_offset4, ShortRunLowGreyLevelEmphasis_AllDirection_offset4_SD,

ShortRunLowGreyLevelEmphasis_angle0_offset4, ShortRunLowGreyLevelEmphasis_angle45_offset4,

ShortRunLowGreyLevelEmphasis_angle90_offset4, ShortRunLowGreyLevelEmphasis_angle135_offset4,

ShortRunLowGreyLevelEmphasis_AllDirection_offset7, ShortRunLowGreyLevelEmphasis_AllDirection_offset7_SD,

ShortRunLowGreyLevelEmphasis_angle0_offset7, ShortRunLowGreyLevelEmphasis_angle45_offset7,

ShortRunLowGreyLevelEmphasis_angle90_offset7, ShortRunLowGreyLevelEmphasis_angle135_offset7

8) **ShortRunHighGreyLevelEmphasis：**

$$SRHGE(\theta)=\frac{1}{n_{r}}\sum_{j=i}^{N} \sum_{i=1}^{M} \frac{p(i,j,\theta)i^{2}}{j^{2}}$$

*Angle=All Direction, 0, 45, 90, 135, All_SD;

*Offset=1, 4, 7

The 18 features of ShortRunHighGreyLevelEmphasis are extracted:

ShortRunHighGreyLevelEmphasis_AllDirection_offset1, ShortRunHighGreyLevelEmphasis_AllDirection_offset1_SD,

ShortRunHighGreyLevelEmphasis_angle0_offset1, ShortRunHighGreyLevelEmphasis_angle45_offset1,

ShortRunHighGreyLevelEmphasis_angle90_offset1, ShortRunHighGreyLevelEmphasis_angle135_offset1,

ShortRunHighGreyLevelEmphasis_AllDirection_offset4, ShortRunHighGreyLevelEmphasis_AllDirection_offset4_SD,

ShortRunHighGreyLevelEmphasis_angle0_offset4, ShortRunHighGreyLevelEmphasis_angle45_offset4,

ShortRunHighGreyLevelEmphasis_angle90_offset4, ShortRunHighGreyLevelEmphasis_angle135_offset4,

ShortRunHighGreyLevelEmphasis_AllDirection_offset7, ShortRunHighGreyLevelEmphasis_AllDirection_offset7_SD,

ShortRunHighGreyLevelEmphasis_angle0_offset7, ShortRunHighGreyLevelEmphasis_angle45_offset7,

ShortRunHighGreyLevelEmphasis_angle90_offset7, ShortRunHighGreyLevelEmphasis_angle135_offset7

9) **LongRunLowGreyLevelEmphasis:**

$$LRLGE(\theta)=\frac{1}{n_{r}}\sum_{j=i}^{N} \sum_{i=1}^{M} \frac{p(i,j,\theta)j^{2}}{i^{2}}$$

*Angle=All Direction, 0, 45, 90, 135, All_SD;

*Offset=1, 4, 7

The 18 features of LongRunLowGreyLevelEmphasis are extracted:

LongRunLowGreyLevelEmphasis_AllDirection_offset1, LongRunLowGreyLevelEmphasis_AllDirection_offset1_SD,

LongRunLowGreyLevelEmphasis_angle0_offset1, LongRunLowGreyLevelEmphasis_angle45_offset1,

LongRunLowGreyLevelEmphasis_angle90_offset1, LongRunLowGreyLevelEmphasis_angle135_offset1,

LongRunLowGreyLevelEmphasis_AllDirection_offset4, LongRunLowGreyLevelEmphasis_AllDirection_offset4_SD,

LongRunLowGreyLevelEmphasis_angle0_offset4, LongRunLowGreyLevelEmphasis_angle45_offset4,

LongRunLowGreyLevelEmphasis_angle90_offset4, LongRunLowGreyLevelEmphasis_angle135_offset4,

LongRunLowGreyLevelEmphasis_AllDirection_offset7, LongRunLowGreyLevelEmphasis_AllDirection_offset7_SD,

LongRunLowGreyLevelEmphasis_angle0_offset7, LongRunLowGreyLevelEmphasis_angle45_offset7,

LongRunLowGreyLevelEmphasis_angle90_offset7, LongRunLowGreyLevelEmphasis_angle135_offset7

10) **LongRunHighGreyLevelEmphasis:**

$$LRHGE(\theta)=\frac{1}{n_{r}}\sum_{j=i}^{N} \sum_{i=1}^{M} p(i,j,\theta)i^{2}j^{2}$$

where *n_r_* is the total number of runs and *n_p_* is the number of pixels in the image.

*Angle=All Direction, 0, 45, 90, 135, All_SD;

*Offset=1, 4, 7

The 18 features of LongRunHighGreyLevelEmphasis are extracted:

LongRunHighGreyLevelEmphasis_AllDirection_offset1, LongRunHighGreyLevelEmphasis_AllDirection_offset1_SD,

LongRunHighGreyLevelEmphasis_angle0_offset1, LongRunHighGreyLevelEmphasis_angle45_offset1,

LongRunHighGreyLevelEmphasis_angle90_offset1, LongRunHighGreyLevelEmphasis_angle135_offset1,

LongRunHighGreyLevelEmphasis_AllDirection_offset4, LongRunHighGreyLevelEmphasis_AllDirection_offset4_SD,

LongRunHighGreyLevelEmphasis_angle0_offset4, LongRunHighGreyLevelEmphasis_angle45_offset4,

LongRunHighGreyLevelEmphasis_angle90_offset4, LongRunHighGreyLevelEmphasis_angle135_offset4,

LongRunHighGreyLevelEmphasis_AllDirection_offset7, LongRunHighGreyLevelEmphasis_AllDirection_offset7_SD,

LongRunHighGreyLevelEmphasis_angle0_offset7, LongRunHighGreyLevelEmphasis_angle45_offset7,

LongRunHighGreyLevelEmphasis_angle90_offset7, LongRunHighGreyLevelEmphasis_angle135_offset7

**5.6 Grey-level zone size matrix (GLZSM) features**

The grey level Size Zone Matrix (SZM) is the starting point of Thibault matrices. For a texture image f with N grey levels, it is denoted GSf(s, g) and provides a statistical representation by the estimation of a bivariate conditional probability density function of the image distribution values. It is calculated according to the pioneering Run Length Matrix principle: the value of the matrix GSf(s, g) is equal to the number of zones of size s and of grey level g. The resulting matrix has a fixed number of lines equal to N, the number of grey levels, and a dynamic number of columns, determined by the size of the largest zone as well as the size quantization.

The more homogeneous the texture, the wider and flatter the matrix. SZM does not required computation in several directions. However, it has been empirically proved that the degree of grey level quantization still has an important impact on the texture classification performance.

Let P define the GLSZM of a quantized volume V (x,y,z) with isotropic voxel size. *P(i,j)* represents the number of 3D zones of grey-levels *i* and of size j in V , *Ng* represents the pre-defined number of quantized grey-levels set in V, and *Lz* represents the size of the largest zone (of any grey-level) in V. One GLSZM of size *Ng*× *Lz* is computed per volume V by adding up all possible largest zone-sizes, with zones constructed from 26-connected neighbours of the same grey-level in 3D space (one voxel can be part of only one zone). The entry *(i,j)* of the normalized GLSZM is then defined as:

The following quantities are also defined:

## **1) SmallAreaEmphasis:**

## **2) LargeAreaEmphasis:**

## **3) IntensityVariability:**

## **4) SizeZoneVariability:**

## **5) ZonePercentage**

## **6) LowIntensityEmphasis:**

## **7) HighIntensityEmphasis:**

## **8) LowIntensitySmallAreaEmphasis:**

## **9) HighIntensitySmallAreaEmphasis:**

**10) LowIntensityLargeAreaEmphasis:**

## 11) **HighIntensityLargeAreaEmphasis:**

**S6. Detailed descriptions and results of predictive models in the independent testing cohort**

Between March 2022 and April 2023, we collected 17 patients with HCC who underwent CE-MRI examination before initial TACE treatment according to the inclusion and exclusion criteria. On the basis of the mRECIST criterion, the patients for OR and NR were 12 (70.6%) and 5 (29.4%), respectively. Clinical-radiological characteristics and intratumoral and peritumoral radiomics features based on CE-MR images were acquired. The 17 patients were used as an independent testing cohort to validate the performance of predictive models constructed by the training cohort. There were no significant differences in the AUCs of the predictive models between the training and the independent testing cohorts (all *P* > 0.05). The model performances of the independent testing cohort were moderate to excellent in general (AUC values: from 0.717 to 0.917) except for PTR (5 mm) and PTR (10 mm) models. However, the sample size of the most recent patients included in the study was small, which may introduce a certain bias and limit the reproducibility of the model. We will try to collect a larger cohort population to validate the robustness and reproducibility of the predictive models and further reinforce the conclusions of our study during the next research.

**Discrimination performance of different predictive models in the training, the validation, and the independent testing cohorts**

| Model | | AUC (95% CI) | Accuracy | Sensitivity | Specificity | *P* value |
| --- | --- | --- | --- | --- | --- | --- |
| Clinical-radiological model | Training | 0.789 (0.709 - 0.863) | 0.729 | 0.556 | 0.882 | 0.928 |
|  | Validation | 0.782 (0.660 - 0.902) | 0.714 | 0.600 | 0.818 |  |
|  | Testing | 0.800 (0.578 - 1.000) | 0.588 | 1.000 | 0.417 |  |
| TR model | Training | 0.836 (0.763 - 0.903) | 0.771 | 0.778 | 0.765 | 0.669 |
|  | Validation | 0.820 (0.705 - 0.917) | 0.714 | 0.750 | 0.682 |  |
|  | Testing | 0.783 (0.558 - 1.000) | 0.765 | 1.000 | 0.667 |  |
| PTR (3 mm) model | Training | 0.817 (0.740 - 0.881) | 0.750 | 0.889 | 0.627 | 0.912 |
|  | Validation | 0.823 (0.701 - 0.927) | 0.714 | 0.800 | 0.636 |  |
|  | Testing | 0.800 (0.521 - 1.000) | 0.882 | 0.600 | 1.000 |  |
| PTR (5 mm) model | Training | 0.810 (0.739 - 0.880) | 0.771 | 0.800 | 0.745 | 0.171 |
|  | Validation | 0.823 (0.711 - 0.924) | 0.714 | 0.900 | 0.545 |  |
|  | Testing | 0.567 (0.243 - 0.891) | 0.647 | 0.600 | 0.667 |  |
| PTR (10 mm) model | Training | 0.852 (0.785 - 0.911) | 0.760 | 0.822 | 0.706 | 0.151 |
|  | Validation | 0.793 (0.673 - 0.903) | 0.762 | 0.900 | 0.636 |  |
|  | Testing | 0.567 (0.202 - 0.932) | 0.706 | 0.600 | 0.750 |  |
| T-PTR (3 mm) model | Training | 0.884 (0.821 - 0.936) | 0.812 | 0.822 | 0.804 | 0.772 |
|  | Validation | 0.911 (0.825 - 0.975) | 0.810 | 0.800 | 0.818 |  |
|  | Testing | 0.850 (0.633 - 1.000) | 0.882 | 0.800 | 0.917 |  |
| T-PTR (5 mm) model | Training | 0.883 (0.822 - 0.934) | 0.823 | 0.733 | 0.902 | 0.355 |
|  | Validation | 0.909 (0.817 - 0.982) | 0.810 | 0.650 | 0.955 |  |
|  | Testing | 0.767 (0.536 - 0.997) | 0.706 | 1.000 | 0.583 |  |
| T-PTR (10 mm) model | Training | 0.892 (0.834 - 0.942) | 0.844 | 0.800 | 0.882 | 0.266 |
|  | Validation | 0.895 (0.815 - 0.964) | 0.786 | 0.750 | 0.818 |  |
|  | Testing | 0.717 (0.426 - 1.000) | 0.765 | 0.600 | 0.833 |  |
| Combined nomogram | Training | 0.910 (0.854 - 0.958) | 0.844 | 0.822 | 0.863 | 0.936 |
|  | Validation | 0.918 (0.831 - 0.986) | 0.857 | 0.800 | 0.909 |  |
|  | Testing | 0.917 (0.775 - 1.000) | 0.882 | 1.000 | 0.833 |  |

TR, intratumoral region; PTR, peritumoral region; T-PTR, intratumoral and peritumoral region; AUC, area under the curve; CI, confidence interval. *P* value: the comparison in the AUC values between the training cohort and the independent testing cohort was assessed by the Delong’s test.

**S7. The formulae of the radiomics score**

| Rad-score | Calculation formulae |
| --- | --- |
| Rad-score_TR | =-0.443  +0.558×A_Variance  +0.247×A_histogramEnergy  -0.123×A_skewness  +0.574×A_uniformity  +0.271×A_ClusterShade_AllDirection_offset4_SD  -0.480×A_GLCMEntropy_angle45_offset7  +1.309×A_GLCMEntropy_angle90_offset1  -0.688×A_HaralickCorrelation_angle90_offset1  -0.056×A_InverseDifferenceMoment_angle45_offset4  -0.673×A_LongRunHighGreyLevelEmphasis_AllDirection_offset7_SD  +0.224×A_ShortRunEmphasis_angle90_offset1  -0.353×A_ShortRunHighGreyLevelEmphasis_AllDirection_offset1_SD  +0.759×A_ShortRunHighGreyLevelEmphasis_AllDirection_offset7_SD  -0.489×A_LowIntensityEmphasis  -2.163×A_ZonePercentage  -0.822×V_GLCMEntropy_angle135_offset7  -0.523×V_Inertia_angle90_offset4  +0.258×V_LongRunEmphasis_angle0_offset7  +0.802×V_LongRunHighGreyLevelEmphasis_AllDirection_offset4_SD  +0.426×V_ShortRunEmphasis_angle0_offset1  -0.265×V_ShortRunEmphasis_angle0_offset4  -1.162×V_ShortRunHighGreyLevelEmphasis_AllDirection_offset4_SD  -0.065×D_InverseDifferenceMoment_angle135_offset1  -0.739×D_InverseDifferenceMoment_angle135_offset4  -1.300×D_SurfaceVolumeRatio |
| Rad-score_PTR (3 mm) | =-0.100  -0.190×A_VoxelValueSum  +0.681×A_HaralickCorrelation_AllDirection_offset4_SD  +0.555×A_differenceEntropy  -0.142×A_LongRunHighGreyLevelEmphasis_angle0_offset7  +0.556×A_Compactness2  +1.672×A_MinorAxisLength  +0.126×A_HighIntensityLargeAreaEmphasis  +0.213×A_LowIntensityEmphasis  -0.675×V_HaralickCorrelation_angle90_offset4  -0.011×V_Compactness2  -0.020×D_GLCMEntropy_angle135_offset4  +0.173×D_sumAverage  +0.091×D_IntensityVariability  -0.227×D_ZonePercentage |
| Rad-score_PTR (5 mm) | =-0.139  -0.763×A_VoxelValueSum  +0.684×A_Correlation_angle45_offset1  +1.575×A_Inertia_angle135_offset1  -0.498×A_Inertia_angle90_offset7  -0.215×A_ShortRunEmphasis_angle135_offset7  +0.656×A_Compactness2  +0.894×A_SphericalDisproportion  +0.062×V_GLCMEntropy_AllDirection_offset7_SD  +0.458×V_GLCMEntropy_angle135_offset7  +0.858×V_Compactness1-0.121×V_Compactness2  +0.194×V_LowIntensityLargeAreaEmphasis  -0.161×D_Correlation_angle45_offset1  +0.168×D_HaralickCorrelation_angle135_offset4  +0.034×D_Inertia_angle0_offset7-0.556×D_Inertia_angle45_offset1  -0.575×D_InverseDifferenceMoment_angle0_offset1 |
| Rad-score_PTR (10 mm) | =-0.379  -0.478×A_skewness  +0.262×A_GLCMEntropy_AllDirection_offset4  +0.543×A_Inertia_angle135_offset7  -0.030×A_InverseDifferenceMoment_angle135_offset7  -0.603×A_HighGreyLevelRunEmphasis_AllDirection_offset7_SD  -1.379×A_ShortRunHighGreyLevelEmphasis_AllDirection_offset4_SD  +0.634×A_Compactness2  -0.450×A_SphericalDisproportion  -0.148×A_SurfaceArea  +0.440×V_Variance-0.239×V_Inertia_angle135_offset7  +1.590×V_InverseDifferenceMoment_angle0_offset1  +0.024×V_RunLengthNonuniformity_AllDirection_offset1_SD  +1.163×V_ShortRunEmphasis_angle0_offset1  -0.863×V_ShortRunHighGreyLevelEmphasis_AllDirection_offset4_SD  -0.361×V_ShortRunHighGreyLevelEmphasis_AllDirection_offset7_SD  +2.247×V_Maximum3Ddiameter  +0.184×D_sumAverage+0.376×D_ShortRunEmphasis_angle90_offset1  -0.485×D_ShortRunHighGreyLevelEmphasis_AllDirection_offset1_SD  +0.523×D_ShortRunHighGreyLevelEmphasis_AllDirection_offset4_SD |
| Rad-score_T-PTR (3 mm) | = -0.077+0.759×rad-score_TR+0.716×rad-score_PTR (3 mm) |
| Rad-score_T-PTR (5 mm) | =-0.230+0.885×rad-score_TR+0.766×rad-score_PTR (5 mm) |
| Rad-score_T-PTR (10 mm) | =0.060+0.522×rad-score_TR+0.825×rad-score_PTR (10 mm) |

Rad-score, radiomics score; TR, intratumoral region; PTR, peritumoral region; T-PTR, intratumoral and peritumoral region; AP, arterial phase; PVP, portal venous phase; DP, delayed phase; GLCM, grey level co-occurrence matrix.

**S8. ROC curves of the seven radiomics models in the training (A) and validation (B) cohorts**

**S9. Delong’s test of different predictive models in the training and validation cohorts**

| Validation  Training | TR model | PTR (3 mm) model | PTR (5 mm) model | PTR (10 mm) model | T-PTR (3 mm) model | T-PTR (5 mm) model | T-PTR (10 mm) model | Clinical-  radiological model | Combined nomogram |
| --- | --- | --- | --- | --- | --- | --- | --- | --- | --- |
| TR model | — | 0.981 | 0.981 | 0.779 | 0.258 | 0.284 | 0.354 | 0.697 | 0.235 |
| PTR (3 mm) model | 0.742 | — | 1.000 | 0.768 | 0.290 | 0.316 | 0.389 | 0.688 | 0.265 |
| PTR (5 mm) model | 0.673 | 0.922 | — | 0.761 | 0.268 | 0.295 | 0.367 | 0.679 | 0.244 |
| PTR (10 mm) model | 0.777 | 0.535 | 0.478 | — | 0.166 | 0.186 | 0.233 | 0.912 | 0.151 |
| T-PTR (3 mm) model | 0.372 | 0.222 | 0.195 | 0.526 | — | 0.973 | 0.807 | 0.139 | 0.918 |
| T-PTR (5 mm) model | 0.379 | 0.226 | 0.198 | 0.537 | 0.978 | — | 0.842 | 0.157 | 0.896 |
| T-PTR (10 mm) model | 0.295 | 0.169 | 0.148 | 0.427 | 0.877 | 0.854 | — | 0.197 | 0.736 |
| Clinical-radiological model | 0.436 | 0.656 | 0.734 | 0.279 | 0.094 | 0.094 | 0.067 | — | 0.127 |
| Combined nomogram | 0.153 | 0.080 | 0.070 | 0.232 | 0.577 | 0.551 | 0.683 | 0.028 | — |

TR, intratumoral region; PTR, peritumoral region; T-PTR, intratumoral and peritumoral region.

**S10. Stratified predictive performance on the subgroups of different inclusion time**

| Model | AUC (95% CI) | *P* value |
| --- | --- | --- |
| Clinical-radiological model |  | 0.464^a^ |
| Subgroup 1 | 0.819 (0.720 - 0.917) | 0.641^b^ |
| Subgroup 2 | 0.764 (0.657 - 0.871) | 0.704^c^ |
| Overall cohort | 0.789 (0.716 - 0.863) |  |
| TR model |  | 0.814^a^ |
| Subgroup 1 | 0.847 (0.748 - 0.945) | 0.860^b^ |
| Subgroup 2 | 0.830 (0.739 - 0.921) | 0.924^c^ |
| Overall cohort | 0.836 (0.770 - 0.902) |  |
| PTR (3 mm) model |  | 0.278^a^ |
| Subgroup 1 | 0.857 (0.764 - 0.951) | 0.507^b^ |
| Subgroup 2 | 0.778 (0.671 - 0.886) | 0.551^c^ |
| Overall cohort | 0.818 (0.747 - 0.888) |  |
| PTR (5 mm) model |  | 0.703^a^ |
| Subgroup 1 | 0.813 (0.710 - 0.916) | 0.953^b^ |
| Subgroup 2 | 0.784 (0.671 - 0.896) | 0.704^c^ |
| Overall cohort | 0.810 (0.738 - 0.882) |  |
| PTR (10 mm) model |  | 0.586^a^ |
| Subgroup 1 | 0.854 (0.764 - 0.944) | 0.750^b^ |
| Subgroup 2 | 0.818 (0.722 - 0.913) | 0.754^c^ |
| Overall cohort | 0.836 (0.771 - 0.901) |  |
| T-PTR (3 mm) model |  | 0.472^a^ |
| Subgroup 1 | 0.911 (0.836 - 0.987) | 0.623^b^ |
| Subgroup 2 | 0.870 (0.787 - 0.953) | 0.729^c^ |
| Overall cohort | 0.888 (0.832 - 0.943) |  |
| T-PTR (5 mm) model |  | 0.859^a^ |
| Subgroup 1 | 0.904 (0.835 - 0.974) | 0.833^b^ |
| Subgroup 2 | 0.895 (0.823 - 0.967) | 0.996^c^ |
| Overall cohort | 0.895 (0.843 - 0.946) |  |
| T-PTR (10 mm) model |  | 0.979^a^ |
| Subgroup 1 | 0.894 (0.815 - 0.974) | 0.972^b^ |
| Subgroup 2 | 0.896 (0.825 - 0.967) | 0.995^c^ |
| Overall cohort | 0.896 (0.845 - 0.947) |  |
| Combined nomogram |  | 0.359^a^ |
| Subgroup 1 | 0.934 (0.872 - 0.995) | 0.503^b^ |
| Subgroup 2 | 0.886 (0.806 - 0.966) | 0.687^c^ |
| Overall cohort | 0.906 (0.853 - 0.958) |  |

TR, intratumoral region; PTR, peritumoral region; T-PTR, intratumoral and peritumoral region; AUC, area under the curve; CI, confidence interval. Subgroup 1: patients were included between April 2008 and June 2015; subgroup 2: patients were included between July 2015 and February 2022. ^a^ *P* value: the comparison in the AUC values between the subgroups of inclusion time was assessed by the Delong’s test. ^b^ *P* value: the comparison in the AUC values between the subgroup 1 and the overall cohort was assessed by the Delong’s test. ^c^ *P* value: the comparison in the AUC values between the subgroup 2 and the overall cohort was assessed by the Delong’s test.
